# Exploring the Effect of High-Energy Heavy Ion Beam on Rice Genome: Transposon Activation

**DOI:** 10.3390/genes14122178

**Published:** 2023-12-04

**Authors:** Xiaoting Wen, Jingpeng Li, Fu Yang, Xin Zhang, Yiwei Li

**Affiliations:** 1 Key Laboratory of Soybean Molecular Design and Breeding, Northeast Institute of Geography and Agroecology, Chinese Academy of Sciences, Changchun 130102, China; wenxiaoting@iga.ac.cn (X.W.); yangfu@iga.ac.cn (F.Y.); zhangxin183@mails.ucas.ac.cn (X.Z.); liyiwei211@mails.ucas.ac.cn (Y.L.); 2 University of Chinese Academy of Sciences, Beijing 100049, China; 3 Jilin Provincial Laboratory of Crop Germplasm Resources, Changchun 130299, China

**Keywords:** high-energy heavy ion beam, whole-genome sequencing, transposon, rice, mutant

## Abstract

High-energy heavy ion beams are a new type of physical mutagen that can produce a wide range of phenotypic variations. In order to understand the mechanism of high-energy heavy ion beams, we resequenced the whole genome of individual plants with obvious phenotypic variations in rice. The sequence alignment results revealed a large number of SNPs and InDels, as well as genetic variations related to grain type and heading date. The distribution of SNP and InDel on chromosomes is random, but they often occur in the up/downstream regions and the intergenic region. Mutagenesis can cause changes in transposons such as *Dasheng*, *mPing*, *Osr13* and *RIRE2*, affecting the stability of the genome. This study obtained the major gene mutation types, discovered differentially active transposons, screened out gene variants related to phenotype, and explored the mechanism of high-energy heavy ion beam radiation on rice genes.

## 1. Introduction

Rice is a fundamental staple food crop that feeds a significant portion of the global population and holds a crucial position in ensuring food security on a global scale [1]. To enhance the genetic diversity of rice, novel rice varieties have been generated through diverse breeding approaches. Among these techniques, mutagenesis stands as a prominent method widely employed to induce genetic variations in rice crops [2]. By utilizing mutagenesis, rice mutants with improved yield potential and superior quality characteristics can be selectively screened and identified, facilitating the development of superior rice cultivars [3].

Artificial mutations can be mainly classified into chemical mutagens and physical mutagens [4]. Ethyl methane sulfonate (EMS) is a widely used chemical mutagen, which can preferentially alkylate guanine (G) residues and mainly induces GC > AT transition (93%) [5]. However, chemical mutagens are usually hazardous, and treatments of chemical mutagens are time-consuming. In contrast to chemical mutagens, physical mutagenesis has a stronger penetration ability, and treatments by physical mutagenesis are usually fast, leaving no hazardous waste [3]. γ rays (GRs) and ion beams are widely used in physical mutagens; all of them are ionizing radiation carrying more energy than nonionizing radiation. However, GR and ion beam irradiation have different energy deposition modes [6]. Heavy ion beams could densely deposit their energy [7], while GRs sparsely deposit their energy in a large target volume [8] so that the linear energy transfer (LET) of GRs is significantly lower than that of ion beams [9,10]. High-energy heavy ion beams are a type of ion beam that has a higher linear energy transfer and, therefore, higher relative biological effectiveness (RBE) and a wider mutational spectrum [11].

The mutant effects of ion irradiation on plant DNA molecules primarily encompass two categories: direct damage and indirect damage [12,13,14]. Direct damage occurs when ions directly act on DNA, leading to double-strand breakage, which can result in base pair substitutions, insertions/deletions, and chromosome rearrangements, consequently serving as a significant source of genomic variation [15]. Indirect damage primarily involves two aspects. Firstly, ionizing radiation leads to the decomposition of H_2_O into •OH and •H [16], with •OH directly reacting with guanine (the most easily oxidized base) to produce 8-oxo-dG, which can mismatch with adenine [17]. Additionally, active oxygen can directly target proteins, leading to a loss of protein function [18]. Secondly, high-energy heavy ion beam irradiation has been shown to induce hypomethylation of CG sites [19], which can activate transposon expression and trigger genomic variation [20]. Maekawa et al. utilized carbon and helium ion beams to irradiate rice and identified a variegated yellow leaf mutant in the progeny, suggesting the activation of transposons [19]. However, no further investigation was conducted in this regard.

The rapid advancements in sequencing technology and their applications have greatly enhanced our understanding of the mutagenic effect of mutagens. Whole-genome resequencing (WGS) has emerged as a powerful tool for detecting gene mutations [21] by comprehensively sequencing and comparing the genomes of different individuals with known reference sequences. The application of WGS to rice mutants generated by carbon ion beams and γ ray irradiation revealed that the most common types of mutations include single base substitutions, insertions, deletions, and polynucleotide mutations [6]. For instance, Morita et al. successfully obtained a grain elongation mutant by using argon (Ar) ion beam mutagenesis, and WGS analysis revealed that it was the result of a single base mutation of the LIN1 [22], which could potentially be utilized for genetic transformation and gene editing purpose. Thus, WGS technology significantly enhanced our comprehensive understanding of mutagen-induced mutation.

In this study, we performed whole-genome resequencing on rice mutants with obvious variations in heading stage and grain type induced by high-energy heavy ion beams. Analyzing gene variation types and transposon differences, it was speculated that the activation of transposons by mutagenesis causes changes in the rice genome, which in turn leads to phenotypic changes in rice mutants. It may provide new ideas for revealing the mutagenesis mechanism of high-energy heavy ion beams.

## 2. Materials and Methods

### 2.1. Plant Materials

In March 2016, we used the Lanzhou Heavy Ion Research Facility (HIRFL) at an energy of 80 MeVu^−1^ to irradiate the 200 seed embryos of Jijing 809 (*O. sativa* L. *japonica*). In April 2016, the irradiated seeds (M_0_) were sowed at Northeast Institute of Geography and Agroecology, Chinese Academy of Sciences, and in M_1_, individually collected. In November of the same year, additional generations were carried out at the Hainan Breeding Base. The M_3_, with excellent comprehensive traits and significant changes in agronomic traits such as heading date and grain shape, were screened, and stable M_6_ mutant lines J6002, J6005, and J6008 were obtained by inbred for multiple generations. After harvesting each variety in M_6_ generation, the grain type and thousand-grain weight of rice mutants and wild-type were measured using the SC-G automatic seeds test and thousand kernels weighter, with each variety measured three times. The heading time was recorded when the rice was 50% heading. The significance of seed data and heading date was analyzed using the software R (Version 4.1.3).

### 2.2. Whole-Genome Sequencing and Data Analysis

The fresh leaves of the top third leaf of each variety were collected respectively, and the samples were immediately frozen in liquid nitrogen for genome resequencing. All samples were sent to Beijing Novogene Bioinformatics Company for library construction and Illumina NovaSeq6000 platform sequencing, and the sequencing strategy was 150 bp paired-end sequencing [23]. The raw data were filtered by Trimmomatic (version 0.39) to remove adapters and low-quality bases using the following parameters: LEADING:5, TRAILING:5, HEADCROP:10, MINLEN:75. The filtered data were aligned to the rice reference genome (MSU7.0) using BWA with default parameters [24]. The aligned depth and other mapping metrics were collected using Picard-Tools [25]. Sequence variations, including SNPs and InDels, were called using SAMtools (version 1.15.1) and BCFtools (version 1.15) [25,26]. The raw sequence variations were filtered using VCFtools (version 0.1.16) [27] with depth of >4 and GQ > 20. Variants between each mutant line and the donor line were extracted using BCFtools and then summarized using RTGtools [28]. According to the annotation information of Nipponbare reference genome, the effects of SNPs and InDels for each sample were annotated using SnpEff (version 5.0c) [29]. The copy number variations of chromosome fragments in each sample were detected using CNVnator (version 0.4.1). The transposon insertion polymorphisms among different genotypes were identified using TEFLoN [30].

## 3. Results

### 3.1. Phenotypic Variation in Carbon Ion Beam (CIB)-Induced Mutant Lines

**Figure 1:**
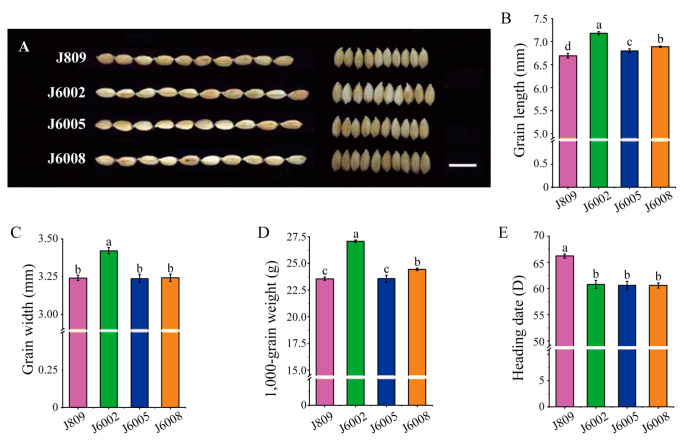
Variations in heading date and grain traits in mutant lines: (**A**) the grain shape of mutants and J809, scale bar: 1 cm; (**B**) differences in grain length in J809, J6002, J6005, and J608; (**C**) differences in grain width in J809, J6002, J6005, and J608; (**D**) differences in thousand-grain weight in J809, J6002, J6005, and J608; (**E**) differences in heading date in J809, J6002, J6005, and J608. Data are the mean ± SD of three biological replicates. The same letter in bars within each figure indicates no significant difference at 0.05 significance level based on Duncan’s multiple range test, and vice versa.

Three mutant lines, J6002, J6005, and J6008, were selected from a large collection of mutants of J809 through high-energy heavy ion beam irradiation treatment. The three mutant lines showed varied phenotypes in heading date, grain weight, and grain shape compared with the donor genotype J809 (Figure 1). The three mutant lines showed significantly longer grain length than J809 (Figure 1B). In addition, the grain width of J6002 was significantly larger than J809, whereas the grain widths of J6005 and J6008 were not statistically different from J809 (Figure 1C). The grain weight was significantly higher in mutant lines than in the donor genotype except for J6005, which showed a similar 1000-grain weight to J809 (Figure 1D). We also found that the heading dates of J6002, J6005, and J6008 were earlier than J809 by 5–7 days (Figure 1E).

### 3.2. Genome-Wide Sequence Variations in Three CIB-Induced Mutant Lines

By sequencing the M_6_ mutant, an average of 96 million total reads were generated in each sample. After that, all the samples were mapped to the reference genome of Nipponbare; the mapping rate of each sample was 99%, and the proportion of Q20 in each sample was above 95%. In sequence alignment, the mean coverage of bases was within 23~30 in each genome. In each sample, the 1× base coverage rate was the upper 93%, and the 5× base coverage rate was at least 91% (Table S1).

**Figure 2:**
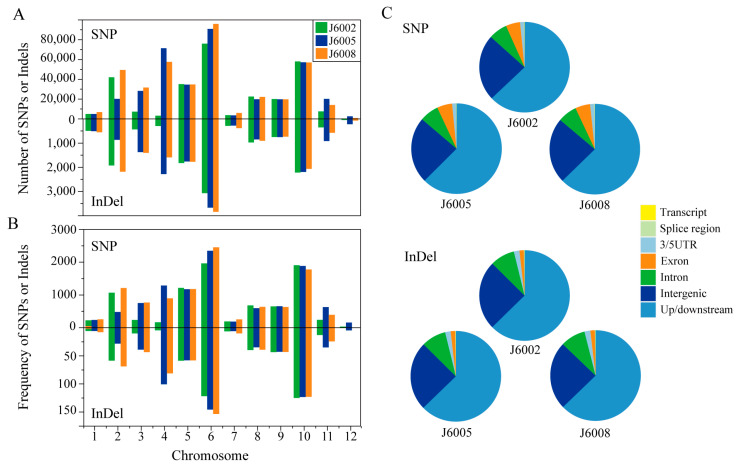
Distribution of SNPs and InDels. Note: (**A**) number of SNPs and InDels on each chromosome; (**B**) rate (variations per Mb) of SNP and InDel on each chromosome; (**C**) functional distribution of SNP and InDel in the genome.

There were 280,848–395,877 single nucleotide polymorphisms (SNPs), and 12,633–16,049 insertion/deletions (InDels) were identified in the three CIB-induced mutants (Figure 2A, Table S2). The overall Ts/Tv ratios of SNPs were 2.5–2.6, and the number of InDels decreased along with InDel length (Figure S1A,B). We investigated the number and rate (variations per Mb) of SNPs and InDels on each chromosome and found that the number and rate of genomic variations were unevenly distributed among chromosomes and that the three mutants showed similar chromosomal distribution patterns (Figure 2). There were more SNPs and InDels on chromosome 6 than on other chromosomes in all mutants; meanwhile, the genomic variations on chromosome 12 were the fewest among all chromosomes (Figure 2A). The distribution of variation rates on chromosomes was similar to the number distribution in all mutations except J6002, in which chromosome 10 showed the highest rate of genomic variants (Figure 2B). We further explored the relationship between variation rates and various chromosome features, including chromosome length, contents of TE and non-TE genes, and CG content; however, none of them was significant (Figure S2). To further address the functional distribution of genomic variations, we analyzed the variations around or within genes. We found the majority of variations were located in the up/downstream or intergenic regions. There were more SNPs than InDels found in exons (Figure 2C).

### 3.3. Transposon Activation in Three CIB-Induced Mutant Lines

**Figure 3:**
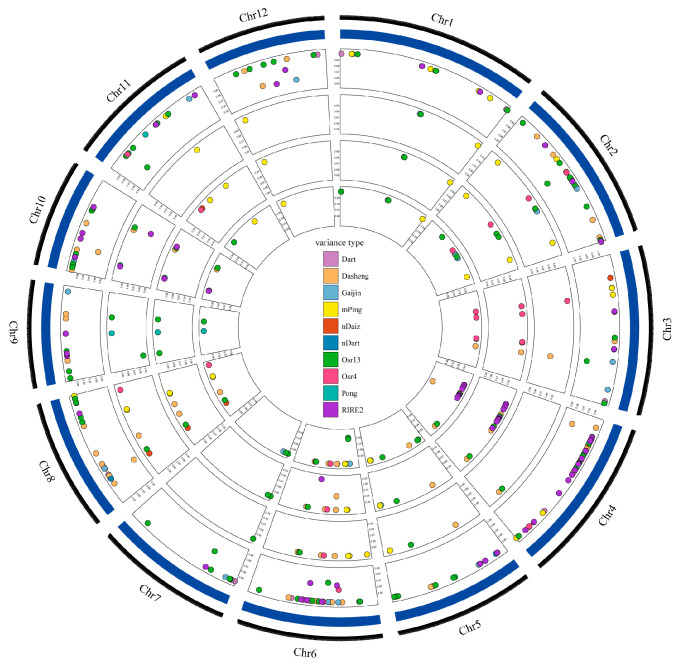
The position distribution of differential transposons. Circles from outside to inside: The first circle refers to the genome sequence information of reference species; the second circle refers to the position information of the differential transposon of J809; the third circle refers to the position information of the differential transposon of J6002; the fourth circle refers to the position information of the differential transposon of J6005; and the fifth circle refers to the position information of the differential transposon of J6008. Different colors represent different transposon types, and the vertical axis values represent the mass information of the variant sites (only variants with mass greater than 0.95 were retained in this study).

In this study, we examined the differences between the parents and mutants in 15 retrotransposons and DNA transposons, which have been reported to be activated or recently activated in rice (Table 1). The results showed that the TE contents were similar among samples, ranging from 2240–2280 (Table 1). *Dasheng*, *RIRE2*, *Osr13*, and *Gaijing* were ubiquitous throughout the genome. Furthermore, by comparing the three mutant lines to the donor genotype, we found both new insertions and eliminations for most TE types except for *Jing*, *Lullaby*, *mGing*, and *Tos17* (Table 2). The number of new insertions was up to 24 for Osr13 in J6008, and the highest number of TE eliminations was 23 for Dasheng in J6005 (Table 2). The types and locations of transposons in J6002, J6005, and J6008 were similar, consisting of the close relationship of the three mutant lines (Figure 3). A few functional genes, including *OsSPCH2* and *OsCPS2*, were found near the transposon insertion polymorphic site, which indicates the activations of transposons in the mutant line might affect the expression of genes that results in phenotype variations (Supplementary Table S2).

### 3.4. Sequence Variations and Functional Genes Related to Phenotypic Changes in Mutants

Sequence variations and functional genes related to phenotypic changes in mutants. Thus, to investigate the possible sequence changes in the three mutant lines, we manually examined the SNPs and InDels located in the known genes related to heading data and grain shape in rice. Ten genes related to flowering (Table 3) and six genes related to grain shape and weight (Table 4) were found to host SNPs and/or InDels between mutants and donor genotypes. After sorting out the variant sites and visualizing in IGV, we found that there were multisite mutations in a large number of genes, and the three mutant lines had the same mutations in a few genes; we speculated that they were derived from the same linage (Table 3 and Table 4, Figure S3). But there was no further verification.

## 4. Discussion

Mutation breeding holds significant importance in germplasm innovation within rice production practices. Among the various mutagenic techniques, high-energy heavy ion beam irradiation has emerged as a novel approach with a broader spectrum of mutations and higher mutation frequency, enabling rapid generation of mutants harboring desired traits [31]. By studying the genomic variation in these mutants, valuable insights can be gained into the nature of genetic changes induced by high-energy heavy ion beams and the underlying mutagenic mechanisms involved.

Upon subjecting the samples to high-energy heavy ion beam irradiation, we successfully obtained mutants with an early heading date of 1 week and large grain size mutants, as depicted in Figure 1. Whole-genome resequencing was performed to unravel the genetic changes in these mutants, revealing a substantial number of SNPs and InDels between the mutant lines and donor genotype. Chromosome-wise analysis showed that the majority of variations were concentrated on Chr.6, while Chr.12 exhibited the lowest frequency of polymorphic sites, with differential variation frequencies observed across different chromosomes (Figure 2A). Further analysis of the distribution of polymorphism sites in relation to chromosomal characteristics (Figure S2) revealed no discernible pattern, indicating the mutation induced by high-energy heavy ion beam is random, but the possibility of mutation hot spots cannot be ruled out. Functional annotation of the variations demonstrated that they predominantly occurred in the up/downstream and intergenic regions, which are known to be susceptible to breakage during cell meiosis and are referred to as “common fragile sites”. Notably, these sites often coincide with active gene regions and transcriptional activity promoter regions [6] (Figure 2B).

The impact of high-energy heavy ion beams on plant genomes encompasses two main aspects: direct DNA damage and the activation of transposable elements, resulting in rice DNA mutations. In this study, we investigated the differences in transposon activity among the mutant lines and identified *mPing* and *Dasheng*, along with *Osr13* and *RIRE2*, as the most prominent transposons (Table 2, Figure 3). *mPing* belongs to miniature inverted-repeats transposable elements (MITEs) [32], whose stability is related to the high-density methylation level of cytosine at the 5’ flanking sequence [33,34,35]. *RIRE2* is an autonomous retrotransposon that contains a small open reading frame (ORF) that is antisense to the genomic RNA transcript, but the function is unknown [36]. Existing studies speculate that *RIRE2* can produce new centromeric satellite repeats, leading to genome amplification [37]. *Dasheng* is a nonautonomous reverse transposon with a size of 5.5 kb to 8.5 kb [38]. Based on the sequence structure of the transposon, the distribution on the chromosome, and the element chimerism in this transpose, it is speculated that the *Dasheng* transposon is activated by *RIRE2* [38]. Previous studies have shown that *Dasheng* belongs to the high-copy-number family and can be activated under normal circumstances [39], which is consistent with our results (Table 1). *Osr13* belongs to the Ty1-copia family and has a very high copy number in rice, which is closely involved in the formation of rice species and may be involved in tissue differentiation [40,41]. High-energy heavy ion beam mutation is a kind of stress that can cause jumping genes transposition, such as *mPing*, *Dasheng*, *RIRE2*, and *Osr13*, probably by inducing hypomethylation of the GC site in the rice genome [19] and ectopic recombination of transposons, therefore impacting genome stability and disrupting gene expression near insertion or inactivation sites (Table S2) [42].

In this study, we screened known genes associated with rice phenotypic changes in all mutants and identified six gene variants related to grain type, namely GW5, qGL5, GW6, GLW7, GW8, and WTG1. Among these genes, GW5 [43], qGL [44], and GLW7 [45] negatively regulate rice grain size, while GW6 [46] and GW8 [47] act as positive regulators. We also detected the mutations in 10 known genes related to heading date, including OsMADS65 [48], SDG725 [49], and Hd3a [50] which promote earlier heading in rice, and RCN2 [51], Ghd2 [52], OsLBD38 [53], OsHAPL1 [54], SDG711 [55], OsCOL15 [56], and Os-GATA28 [57,58] which may promote late flowering phenotypes, although their functions have not been verified. However, some of the gene mutations were not consistent with the observed phenotypic variations, possibly due to gene nonsense mutations. Further investigation of the expression of these genes was not conducted in this study. Grain type and the heading date of rice are quantitative traits controlled by multiple genes, and there is an additive effect among genes, which jointly regulates the phenotypes of these traits [59,60]. However, further studies are needed to explore the interactions among different genes and their impact on rice phenotype. During the comparison of rice genomes, we observed that J6002, J6005, and J6008 share the same variation information in some genes, suggesting that they may originate from the same line.

In this study, we subjected Jijing 809 rice plants to irradiation with high-energy heavy ion beams, resulting in the generation of rice mutants with altered heading dates and grain types. Whole-genome sequencing and sequence comparison revealed the presence of numerous SNPs and InDels in the rice genome, along with gene variations associated with flowering and heading date. Additionally, we observed the activation of transposons, which disrupted the stability of rice genes. This suggests that high-energy heavy ion beams can not only directly induce mutations in the rice genome but also activate transposons, leading to gene mutations in rice.

## Figures and Tables

**Table 1:** Types and copy number of transposons in each genotype.

Category	Type	Super Family	J809	J6008	J6005	J6002
Retrotransposon	*Gypsy*	*Dart*	90	80	80	78
		*Dasheng*	674	661	662	675
		*Gaijin*	184	183	180	181
		*Jing*	37	38	38	38
		*Karma*	4	4	4	5
		*mGing*	16	17	17	17
		*nDaiz*	25	25	27	25
		*nDart*	14	13	14	14
		*RIRE2*	606	605	606	603
	*Copia*	*Lullaby*	5	5	5	5
		*Osr13*	478	477	473	477
		*Osr4*	85	87	85	83
		*Tos17*	8	7	7	8
DNA transposon	*DNA*	*mPing*	42	40	47	44
		*Pong*	5	6	5	5
Total			2273	2248	2250	2258

**Table 2:** Transposon insertions of eliminations in each mutant.

Super Family	Type	J6002	J6005	J6008
*Dart*	(+)	0	0	1
	(−)	8	9	10
*Dasheng*	(+)	10	20	20
	(−)	16	23	21
*Gaijin*	(+)	3	3	8
	(−)	4	4	7
*Jing*	(+)	0	0	0
	(−)	0	0	0
*Lullaby*	(+)	0	0	0
	(−)	0	0	0
*mGing*	(+)	0	0	0
	(−)	0	0	0
*mPing*	(+)	12	12	10
	(−)	6	9	12
*nDaiz*	(+)	1	3	2
	(−)	0	0	0
*nDart*	(+)	0	0	0
	(−)	2	2	1
*Osr13*	(+)	12	20	24
	(−)	7	14	14
*Osr4*	(+)	4	7	7
	(−)	6	6	6
*Pong*	(+)	1	1	1
	(−)	0	1	0
*RIRE2*	(+)	2	14	12
	(−)	9	12	15
*Tos17*	(+)	0	0	0
	(−)	0	0	0

(+) represents the transposon inserted in the mutant compared with the wild-type; (−) represents the transposon missing in the mutant compared with the wild-type.

**Table 3:** Distribution of genetic variation associated with heading date in each mutant.

Mutant Gene	Line	Variant Information
*OsMADS65* (*LOC_Os01g69850*)	J6002	Chr1,40346050, G/A; 40346099, T/C; 40346701, A/C; 40348092, A/C; 40353298, T/C; 40354446, T/C; 40358884, G/A; 40364205, A/G; 40347761, DEL:1;40353958, INS:30;40354148, INS:22
	J6005	Chr1,40346050, G/A; 40346099, T/C; 40346701, A/C; 40348092, A/C; 40353298, T/C; 40354446, T/C; 40358884, G/A; 40364205, A/G; 40347761, DEL:1;40353958, INS:30;40354148, INS:22
	J6008	Chr1,40346050, G/A; 40346099, T/C; 40346701, A/C; 40348092, A/C; 40353298, T/C; 40354446, T/C; 40358884, G/A; 40364205, A/G; 40347761, DEL:1;40353958, INS:30;40354148, INS:22
*RCN2* (*LOC_Os02g32950*)	J6008	Chr2,19567866, C/T; 19567924, A/T; 19568531, C/T; 19568568, C/G; 19568704, G/A; 19568752, G/A; 19568814, A/T; 19567960, DEL:13
*SDG725* (*LOC_Os02g34850*)	J6002	Chr2, 20900113, C/T; 20901575, A/G;20902659, T/C;20902806, G/A;20902923, A/G;20902938, C/T;20903136, A/T;20903813, A/C;20904000, A/G;20904272, C/T;20904360, A/G;20905262, T/C;20905318, T/A;20905609, A/T;20905719, T/C;20905892, C/T;20906077, G/A;20907028, T/G;220902714, DEL:13
	J6008	Chr2,20900113, C/T;20901575, A/G;20902659, T/C;20902806, G/A;20902923, A/G;20902938, C/T; 20903136, A/T;20903813, A/C;20904000, A/G;20904272, C/T;20904360, A/G;20905262, T/C; 20905318, T/A;20905609, A/T;20905719, T/C;20905892, C/T;20906077, G/A;20907028, T/G; 220902714, DEL:18
*Ghd2* (*LOC_Os02g49880*)	J6002	Chr2,30473609, G/A;30474736, T/C
	J6005	Chr2,30473609, G/A; 30474736, T/C
	J6008	Chr2,30473609, G/A; 30474736, T/C
*OsLBD38* (*LOC_Os03g41330*)	J6005	Chr3,22977039, T/C;22977138, A/T;22977558, C/G;22977961, T/G;22978006, G/A;22978008, G/A;22978082, C/G;22978142, A/G;22978156, A/C;22978174, G/A;22979442, A/G;22979762, A/C; 22977280, INS:9; 22977453, DEL14; 22978092, DEL12
	J6008	Chr3, 22977039, T/C;22977138, A/T;22977558, C/G;22977961, T/G;22978006, G/A;22978008, G/A;22978082, C/G;22978142, A/G;22978156, A/C;22978174, G/A;22979442, A/G;22979762, A/C; 22977280, INS:9; 22977453, DEL14; 22978092, DEL12
*OsHAPL1* (*LOC_Os05g41450*)	J6002	Chr5,24277541, T/C;24277683, T/A;24278012, A/G;24278551, T/C
	J6005	Chr5,24277541, T/C; 24277683, T/A; 24278012, A/G; 24278551, T/C; 24280474, A/G; 24280680, T/C
	J6008	Chr5,24277541, T/C; 24277683, T/A; 24278012, A/G; 24278551, T/C
*Hd3a/FT/Ehd3* (*LOC_Os06g06320*)	J6008	Chr6,2942192, C/G
*SDG711* (*LOC_Os06g16390*)	J6005	Chr6,9355225, T/C
	J6008	Chr6,9355225, T/C
*OsCOL15* (*LOC_Os08g42440*)	J6002	Chr8,26793385, T/C; 26793749, T/C; 26794802, A/C; 26795049, A/C; 26795325, T/A; 26795632, A/C; 26795721, C/T; 26796722, C/G; 26792973, DEL,14; 26794149; INS:1; 26797108, DEL:26
	J6005	Chr8,26793385, T/C; 26793749, T/C; 26794802, A/C; 26795049, A/C; 26795325, T/A; 26795632, A/C; 26795721, C/T; 26796722, C/G; 26792973, DEL,26; 26794149; INS:1; 26797108, DEL:6
	J6008	Chr8,26793385, T/C; 26793749, T/C; 26794802, A/C; 26795049, A/C; 26795325, T/A; 26795632, A/C; 26795721, C/T; 26796722, C/G; 26792973, DEL,26; 26794149; INS:1; 26797108, DEL:25
*OsGATA28* (*LOC_Os11g08410*)	J6002	Chr11,4433156, C/T; 4433087, INS:6, 4433137, DEL:11
	J6008	Chr11,4433156, C/T; 4434073, T/A; 4433087, INS:6, 4433137, DEL:19

**Table 4:** Distribution of genetic variation associated with grain type in each mutant.

Mutant Gene	Lines	Variant Information
*GW5* (*LOC_Os05g09520*)	J6002	Chr5, 5365256, G/A; 5366491, INS:20
	J6005	Chr5, 5365256, G/A; 5366491, INS:20
	J6008	Chr5, 5365256, G/A; 5366491, INS:20
*qGL5* (*LOC_Os05g37470*)	J6002	Chr5, 21926820, T/C; 21926832, G/A; 21926911, A/T; 21927295, G/A; 21928349, T/C; 21928809, T/A; 21929398, C/T; 21929564, C/T; 21930387, A/G; 21930730, G/A; 21930884, G/A; 21930900, T/C; 21929789, INS:12
	J6005	Chr5, 21926820, T/C; 21926832, G/A; 21926911, A/T; 21927295, G/A; 21928349, T/C; 21928809, T/A; 21929398, C/T; 21929564, C/T; 21930387, A/G; 21930730, G/A; 21930884, G/A; 21930900, T/C; 21929789, INS:12
	J6008	Chr5, 21926820, T/C; 21926832, G/A; 21926911, A/T; 21927295, G/A; 21928349, T/C; 21928809, T/A; 21929398, C/T; 21929564, C/T; 21930387, A/G; 21930730, G/A; 21930884, G/A; 21930900, T/C; 21929789, INS:12
*GW6* (*LOC_Os06g15620*)	J6005	Chr6, 8847736, A/G; 8847914, A/T; 8848057, A/T; 8848148, G/A; 8848223, A/C; 8848234, A/T; 8848326, C/G; 8848531, T/G; 8848532, C/G; 8848614, C/T; 8848639, C/T; 8848711, G/A; 8848740, A/C; 8848752, T/C; 8847997, INS:2; 8848285, DEL:20; 8848295, DEL:22; 8848308, INS:3; 8848330, DEL:12; 8848601, INS:6; 8848645,DEL:27; 8848655, DEL:23; 8848689, DEL:18; 8848837, DEL:18
	J6008	Chr6, 8847736, A/G; 8847914, A/T; 8848057, A/T; 8848148, G/A; 8848223, A/C; 8848234, A/T; 8848326, C/G; 8848531, T/G; 8848532, C/G; 8848614, C/T; 8848639, C/T; 8848711, G/A; 8848740, A/C; 8848752, T/C; 8847997, INS:2; 8848285, DEL:22; 8848295, DEL:22; 8848308, INS:3; 8848330, DEL:13; 8848601, INS:6; 8848645, DEL:24; 8848654, DEL:23;8848689, DEL:24; 8848837, DEL:18
*GLW7* (*LOC_Os07g32170*)	J6002	Chr7, 19100099, INS:2
	J6005	Chr7, 19100099, INS:2
	J6008	Chr7, 19100099, INS:2
*GW8* (*LOC_Os08g41940*)	J6002	Chr8, 26501258, C/T; 26501458, C/T; 26502275, A/G; 26502571, C/G; 26503084, C/T; 26503280, T/A; 26503323, A/G; 26503397, T/C; 26504208, G/A; 26504323, T/A; 26504422, G/C; 26504599, A/G; 26505008, C/T; 26505025, G/A; 26505046, A/G; 26505387, C/A; 26505658, G/T; 8848285, DEL:20; 26501190, INS:10; 26501542, DEL:16; 26501777, DEL:5; 26501945, INS:15; 26502637, INS:15; 26503416, DEL:13; 26504889, INS:15; 26504922, DEL:14; 26506051, INS:15;
	J6005	Chr8, 26501258, C/T; 26501458, C/T; 26502275, A/G; 26502571, C/G; 26503084, C/T; 26503280, T/A; 26503323, A/G; 26503397, T/C; 26504208, G/A; 26504323, T/A; 26504422, G/C; 26504599, A/G; 26505008, C/T; 26505025, G/A; 26505046, A/G; 26505387, C/A; 26505658, G/T; 26501190, INS:10; 26501542, DEL:17;26501777, DEL:3; 26501945, INS:19; 26502637, INS:12; 26503416, DEL:13; 26504889, INS:17; 26504922, DEL:22; 26506051, INS:16;
	J6008	Chr8, 26501258, C/T; 26501458, C/T; 26502275, A/G; 26502571, C/G; 26503084, C/T; 26503280, T/A; 26503323, A/G; 26503397, T/C; 26504208, G/A; 26504323, T/A; 26504422, G/C; 26504599, A/G; 26505008, C/T; 26505025, G/A; 26505046, A/G; 26505387, C/A; 26505658, G/T; 26501190, INS:10; 26501542, DEL:23; 26501777, DEL:9; 26501945, INS:19; 26502637, INS:18; 26503416, DEL:18; 26504889, INS:14; 26504922, DEL:14; 26506051, INS:18;
*WTG1* (*LOC_Os08g42540*)	J6002	Chr8, 26883121, C/T; 26883146, G/C; 26883932, T/A; 26884169, T/G; 26885532, G/A; 26885992, G/A; 26886283, C/A; 26886650, C/T; 26886715, G/A; 26886719, C/A; 26887098, C/T; 26887145, T/A; 26884796, INS:30; 26886522, DEL:16
	J6005	Chr8, 26883121, C/T; 26883146, G/C; 26883932, T/A; 26884169, T/G; 26885532, G/A; 26885992, G/A; 26886283, C/A; 26886715, G/A; 26886719, C/A; 26884796, INS:25; 26886522, DEL:12
	J6008	Chr8, 26883121, C/T; 26883146, G/C; 26883932, T/A; 26884169, T/G; 26885532, G/A; 26885992, G/A; 26886283, C/A; 26886650, C/T; 26886715, G/A; 26886719, C/A; 26887098, C/T; 26887145, T/A; 26884796, INS:21; 26886522, DEL:13

## Data Availability

The DNA sequencing data for this study was submitted to the NCBI SRA database and can be found under the following accession number: PRJNA971369.

## Supplementary Materials

The following supporting information can be downloaded at: https://www.mdpi.com/article/10.3390/genes14122178/s1. Figure S1: The distribution of SNP mutation type; Figure S2: The distribution of InDels length; Figure S3: Correlation analysis between variation rates and chromosome length (LH), CG content and the contents of transposon element (TE) and non-transposon element (NTE) genes; Table S1: Data analysis of sequencing data in mutants and wild types; Table S2: Total SNPs and Indels in each sample. Gene variation near differential transposons in rice mutants; Gene variation positions related to grain type and heading date.
