# Supplementary material for: Exploring the Effect of High-Energy Heavy Ion Beam on Rice Genome: Transposon Activation
Source: Genes (Basel). 2023 Dec 4;14(12):2178. doi: 10.3390/genes14122178 (PMC10742395; doi:10.3390/genes14122178)
Supplement: Supplementary file 1 [file genes-14-02178-s001.zip › Supplementary_Material 1.pdf]

1 Supplementary Data

Table S1. Data analysis of sequencing data in mutants and wild types

Sample	Total reads	Mapped rate	Q20 value	Mean coverage	Coverage at least 1X	Coverage at least 5X
J809	99491888	0.995007	97.33%	28.465327	94,26%	92.68%
J6002	98499760	0.995078	97.58%	27.947197	94.68%	93.15%
J6005	86218408	0.993698	97.71%	25.739317	93.67%	91.87%
J6008	93434444	0.994276	97.50%	25.981956	93.56%	91.83%

Table S2. Total SNPs and Indels in each sample

Sample	SNPs	Indels
J6002	280848	12633
J6005	373736	15651
J6008	395877	16049

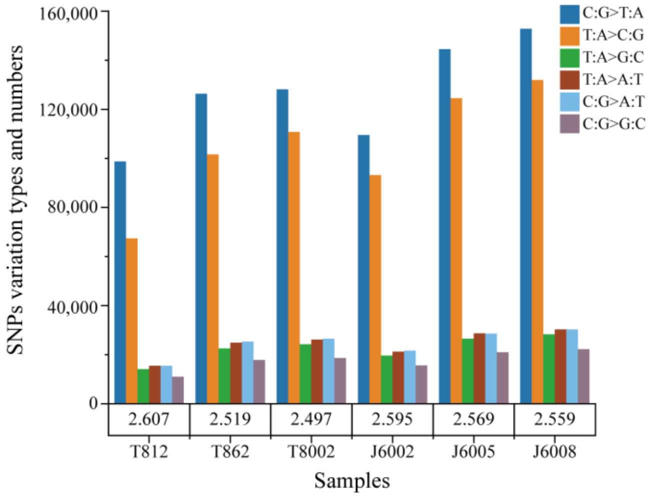

Figure S1. The distribution of SNP mutation type. The number below the bar graph is the ratio of transition and transversion (Ti/Tv).

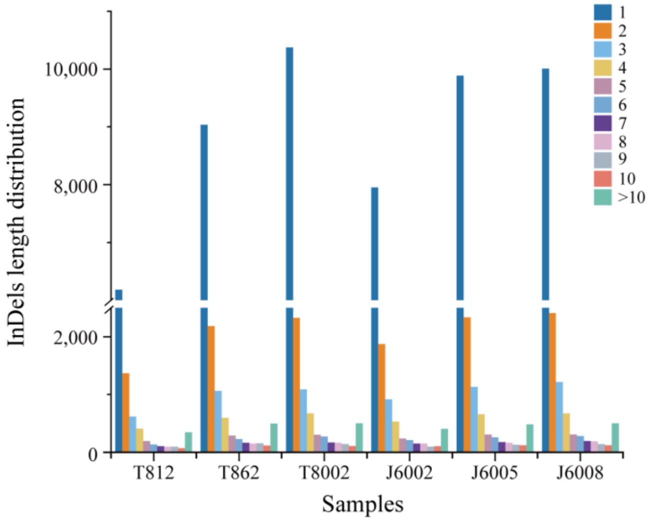

Figure S2. The distribution of InDels length. Different numbers in legend represent the size of the insertion or deletion bases.

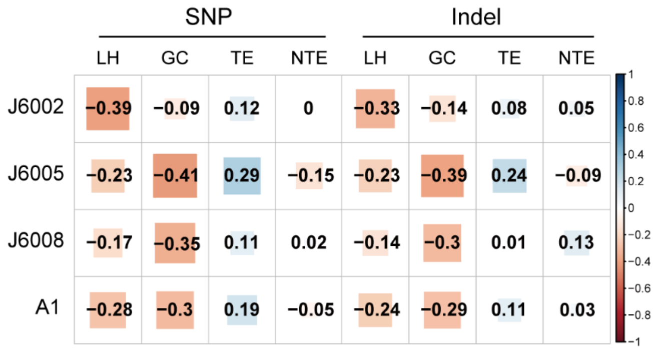

Figure S3. Correlation analysis between variation rates and chromosome length (LH), CG content and the contents of transposon element (TE) and non-transposon element (NTE) genes.
