# Supplementary material for: Exploring the Effect of High-Energy Heavy Ion Beam on Rice Genome: Transposon Activation
Source: Genes (Basel). 2023 Dec 4;14(12):2178. doi: 10.3390/genes14122178 (PMC10742395; doi:10.3390/genes14122178)
Supplement: Supplementary file 1 [file genes-14-02178-s001.zip › Supplementary_Material 2.pdf]

1 Supplementary Data

Gene variation near differential transposons in rice mutants

Sample	Chr	Family	Cluster	BR5	BR3	Gene
J6008	Chr1	Osr13	Copia	284911	292118	LOC_Os01g01560
J6008	Chr1	Osr13	Copia	288372	292296	LOC_Os01g01570
J6008	Chr2	Osr13	Copia	8829119	8829633	LOC_Os02g15680
J6002, J6005, J6008	Chr2	mPing	DNA	8879442	8882660	LOC_Os02g15760
J6008	Chr2	mPing	DNA	13156576	13164110	LOC_Os02g22100
J6002, J6005, J6008	Chr2	Osr4	Copia	17178029	17193550	LOC_Os02g29030
J6002, J6005, J6008	Chr2	Osr4	Copia	17196088	17204631	LOC_Os02g29040
J6008	Chr2	mPing	DNA	19186325	19187087	LOC_Os02g32450
J6008	Chr2	Gaijin	Gypsy	21841059	21847837	LOC_Os02g36210
J6002, J6005, J6008	Chr3	mPing	DNA	7929009	7934005	LOC_Os03g14610
J6002, J6005, J6008	Chr3	Osr4	Copia	12591671	12595636	LOC_Os03g21980
J6008	Chr3	RIRE2	Gypsy	13608896	13612083	LOC_Os03g23980
J6002, J6005, J6008	Chr3	Osr4	Copia	13727326	13738852	LOC_Os03g24180
J6002, J6008	Chr3	Osr13	Copia	22786883	22793943	LOC_Os03g41000
J6008	Chr4	Osr13	Copia	4785358	4789299	LOC_Os04g08780
J6008	Chr4	Osr13	Copia	4791028	4792071	LOC_Os04g08784
J6005, J6008	Chr4	Osr13	Copia	6441984	6442304	LOC_Os04g11760
J6005, J6008	Chr4	Osr13	Copia	6443420	6447642	LOC_Os04g11770
J6005, J6008	Chr4	Osr13	Copia	6448554	6452290	LOC_Os04g11780
J6005, J6008	Chr4	Dasheng	Gypsy	9298246	9301176	LOC_Os04g16990
J6005, J6008	Chr4	Osr13	Copia	11180312	11181920	LOC_Os04g20070
J6005, J6008	Chr4	Osr13	Copia	11538424	11544080	LOC_Os04g20620
J6005, J6008	Chr4	RIRE2	Gypsy	12238259	12241440	LOC_Os04g21640
J6005, J6008	Chr4	RIRE2	Gypsy	12323056	12323515	LOC_Os04g21780
J6005, J6008	Chr4	Dart	Gypsy	26036773	26041443	LOC_Os04g43922
J6005, J6008	Chr4	mPing	DNA	26660002	26661400	LOC_Os04g45060
J6005	Chr4	Dasheng	Gypsy	17180207	17183520	LOC_Os04g28990
J6005	Chr4	Dart	Gypsy	26036773	26041443	LOC_Os04g43922
J6002, J6005	Chr4	mPing	DNA	26660002	26661400	LOC_Os04g45060
J6002, J6005, J6008	Chr4	Osr13	Copia	29417620	29419001	LOC_Os04g49280
J6005	Chr4	Dasheng	Gypsy	31541955	31542752	LOC_Os04g52950
J6005	Chr4	RIRE2	Gypsy	32517267	32522157	LOC_Os04g54660
J6008	Chr5	Osr13	Copia	2233735	2237957	LOC_Os05g04730
J6005, J6008	Chr1	mPing	DNA	23333006	23337594	LOC_Os01g41220
J6008	Chr5	Osr13	Copia	2796435	2797546	LOC_Os05g05620
J6008	Chr5	Osr13	Copia	2798712	2803403	LOC_Os05g05630
J6008	Chr5	Osr13	Copia	2803691	2804985	LOC_Os05g05640
J6002, J6005, J6008	Chr5	RIRE2	Gypsy	7136965	7137684	LOC_Os05g12410
J6002, J6005, J6008	Chr5	Dasheng	Gypsy	7238865	7240419	LOC_Os05g12610

J6005	Chr1	Osr13	Copia	23498443	23502377	LOC_Os01g41510
J6005	Chr1	Osr13	Copia	23503852	23505645	LOC_Os01g41516
J6005	Chr1	Osr13	Copia	23506649	23507760	LOC_Os01g41522
J6002, J6005, J6008	Chr1	Osr13	Copia	23782487	23785775	LOC_Os01g41950
J6002, J6005	Chr5	Dasheng	Gypsy	19631962	19633443	LOC_Os05g33400
J6002, J6005	Chr5	Osr13	Copia	21661349	21665571	LOC_Os05g37070
J6002, J6005	Chr5	Gaijin	Gypsy	27967561	27971088	LOC_Os05g48800
J6005	Chr5	Osr13	Copia	29271238	29274798	LOC_Os05g51000
J6005	Chr5	Osr13	Copia	29276615	29280523	LOC_Os05g51010
J6002, J6008	Chr5	Dasheng	Gypsy	19631962	19633443	LOC_Os05g33400
J6008	Chr5	Osr13	Copia	21661349	21665571	LOC_Os05g37070
J6008	Chr5	Gaijin	Gypsy	27967561	27971088	LOC_Os05g48800
J6008	Chr6	Osr13	Copia	4271134	4272241	LOC_Os06g08590
J6002, J6005, J6008	Chr6	mPing	DNA	6686569	6687306	LOC_Os06g12340
J6005, J6008	Chr6	Dasheng	Gypsy	8506270	8508464	LOC_Os06g15010
J6002, J6005, J6008	Chr6	Osr4	Copia	9364050	9368004	LOC_Os06g16400
J6005, J6008	Chr6	Dasheng	Gypsy	10595341	10595829	LOC_Os06g18680
J6005, J6008	Chr6	Dasheng	Gypsy	10968162	10973326	LOC_Os06g19279
J6008	Chr6	Dart	Gypsy	11237429	11242834	LOC_Os06g19680
J6002, J6005, J6008	Chr6	RIRE2	Gypsy	13394969	13397303	LOC_Os06g22950
J6002, J6005, J6008	Chr6	Osr4	Copia	13704784	13722973	LOC_Os06g23504
J6002, J6005, J6008	Chr6	Dasheng	Gypsy	14555132	14558763	LOC_Os06g24790
J6002, J6005, J6008	Chr6	Dart	Gypsy	16381270	16382990	LOC_Os06g28760
J6002, J6005, J6008	Chr6	RIRE2	Gypsy	16693757	16694669	LOC_Os06g29250
J6002, J6005, J6008	Chr6	Osr4	Copia	17997701	18000860	LOC_Os06g30940
J6002, J6005, J6008	Chr6	Osr13	Copia	20735925	20744696	LOC_Os06g35550
J6005, J6008	Chr7	Osr13	Copia	1260353	1265450	LOC_Os07g03210
J6002, J6005, J6008	Chr8	nDaiz	Gypsy	6319161	6324376	LOC_Os08g10740
J6005, J6008	Chr1	mPing	DNA	38088282	38094202	LOC_Os01g65610
J6002, J6005, J6008	Chr8	Dasheng	Gypsy	13389019	13389835	LOC_Os08g22190
J6005	Chr8	Dasheng	Gypsy	16190684	16193561	LOC_Os08g26620
J6002, J6008	Chr8	Osr4	Copia	26537672	26542925	LOC_Os08g42010
J6002, J6005, J6008	Chr1	mPing	DNA	42638856	42644944	LOC_Os01g73604
J6005	Chr9	Osr13	Copia	3654972	3658804	LOC_Os09g07350
J6005	Chr9	Osr13	Copia	3656769	3665430	LOC_Os09g07360
J6002, J6005, J6008	Chr9	Pong	DNA	11302965	11304308	LOC_Os09g18430
J6002, J6005, J6008	Chr9	Pong	DNA	11304720	11306890	LOC_Os09g18440
J6002, J6005, J6008	Chr10	nDart	Gypsy	1554751	1554957	LOC_Os10g03590
J6002, J6005, J6008	Chr10	Osr13	Copia	1913152	1915827	LOC_Os10g04130
J6008	Chr10	Osr13	Copia	15548856	15550935	LOC_Os10g29960
J6008	Chr10	Osr13	Copia	15552563	15556503	LOC_Os10g29970
J6002, J6005, J6008	Chr10	Dasheng	Gypsy	15663623	15664584	LOC_Os10g30150
J6002, J6005, J6008	Chr10	Osr13	Copia	16486322	16495238	LOC_Os10g31460
J6008	Chr11	Dart	Gypsy	4780136	4782310	LOC_Os11g09000

J6005	Chr11	Osr4	Copia	6218498	6226683	LOC_Os11g11240
J6005	Chr11	nDaiz	Gypsy	6920728	6921120	LOC_Os11g12390
J6005	Chr11	Pong	DNA	11436951	11446266	LOC_Os11g19864
J6002, J6005, J6008	Chr11	Dasheng	Gypsy	18596212	18601900	LOC_Os11g31720
J6002, J6005, J6008	Chr11	mPing	DNA	18774888	18788740	LOC_Os11g31950
