# Supplementary material for: Exploring the Effect of High-Energy Heavy Ion Beam on Rice Genome: Transposon Activation
Source: Genes (Basel). 2023 Dec 4;14(12):2178. doi: 10.3390/genes14122178 (PMC10742395; doi:10.3390/genes14122178)

1 Supplementary Data

Gene variation positions related to grain type and heading date

LOC_Os01g69850

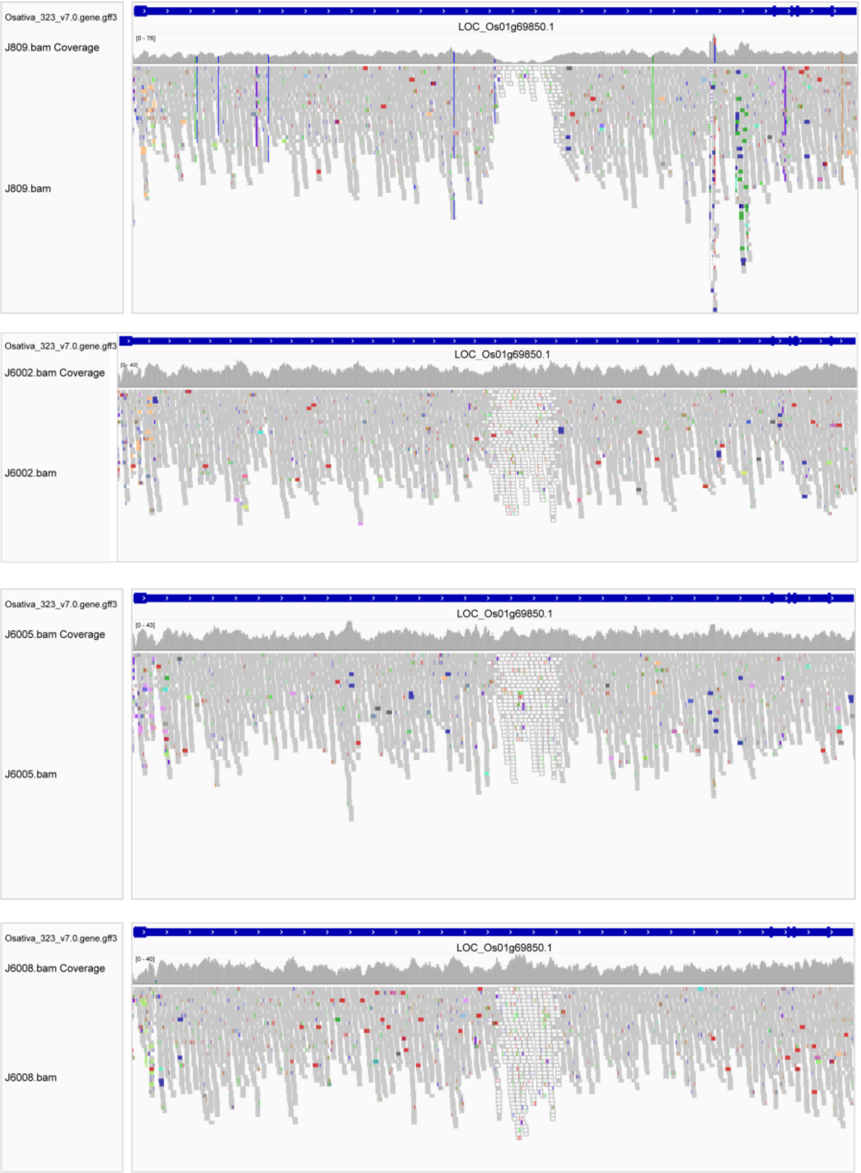

LOC_Os02g32950

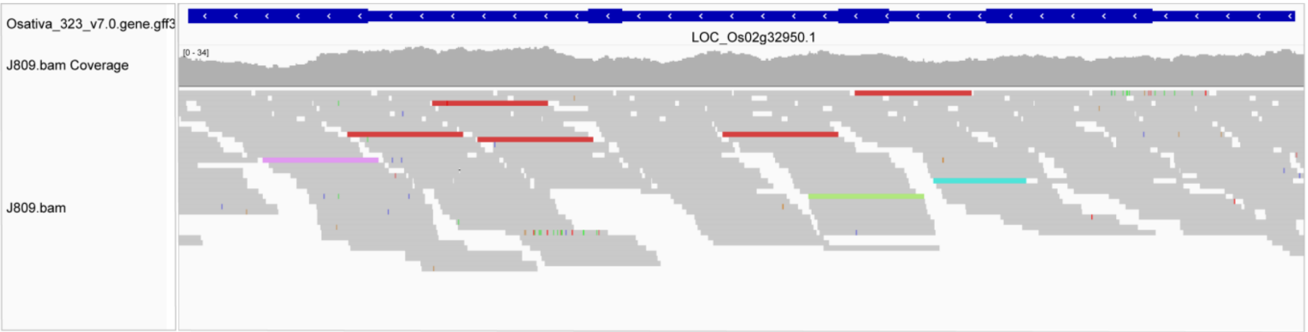
Osativa_323_v7.0.gene.gff3
J809.bam Coverage
J809.bam
LOC_Os02g32950.1
[0 - 34]

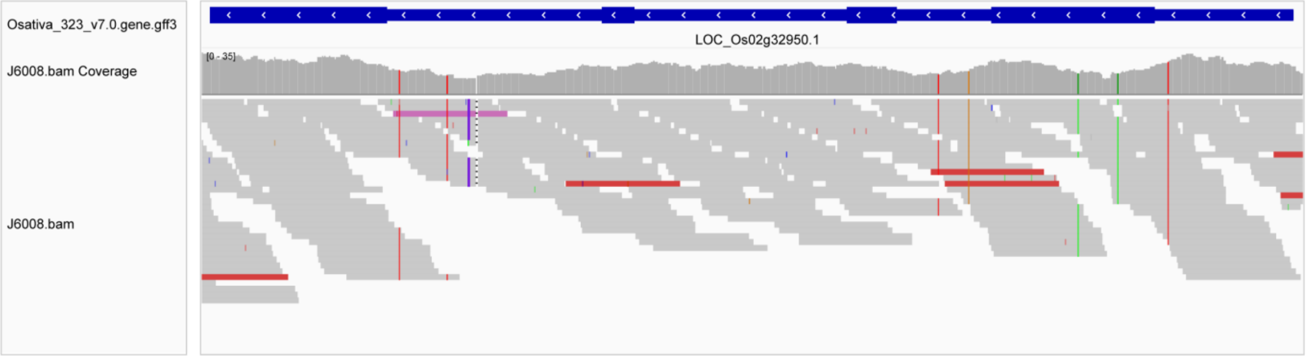
Osativa_323_v7.0.gene.gff3
J6008.bam Coverage
J6008.bam
LOC_Os02g32950.1
[0 - 35]

LOC_Os02g34850

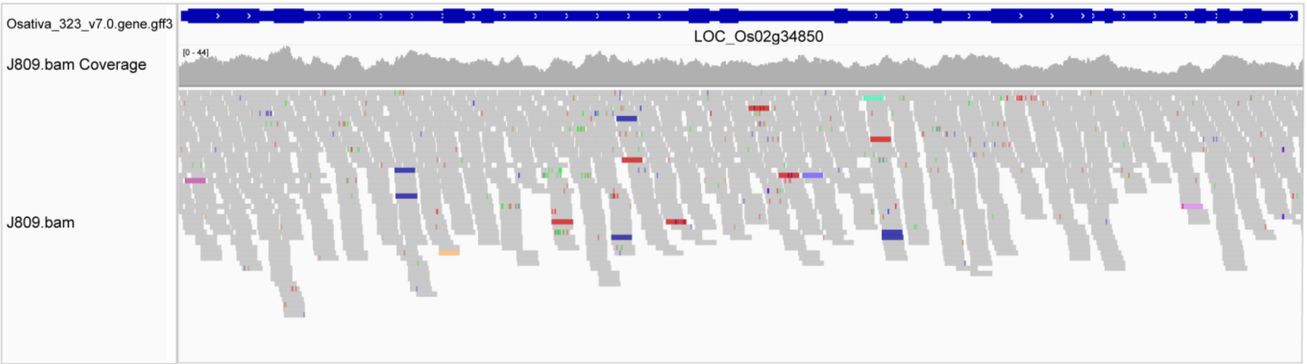

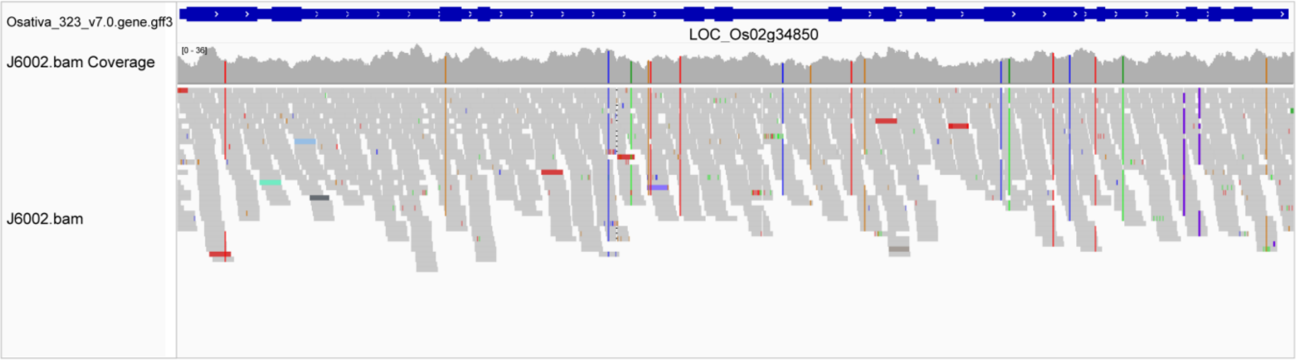

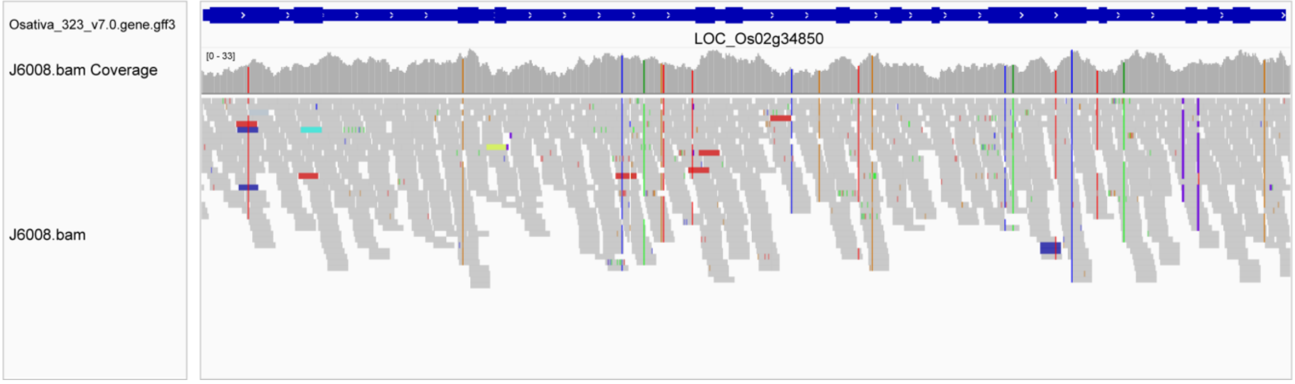

LOC_Os02g49880

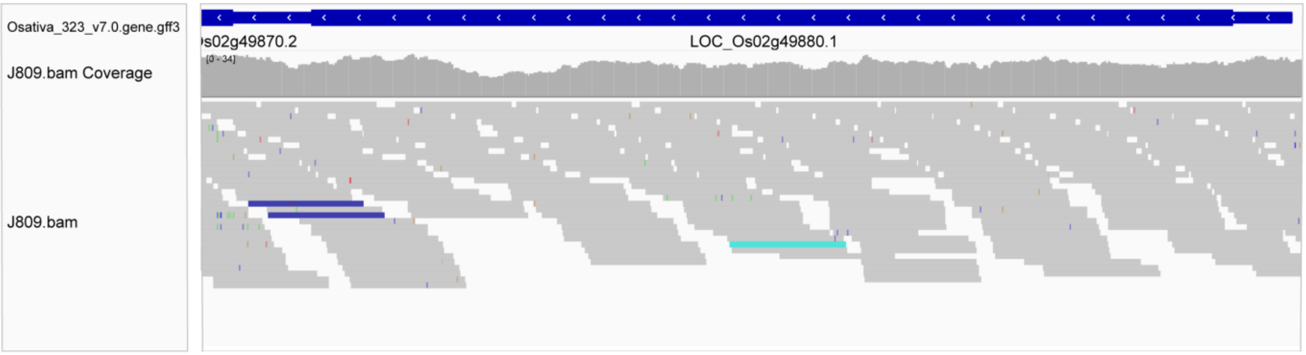

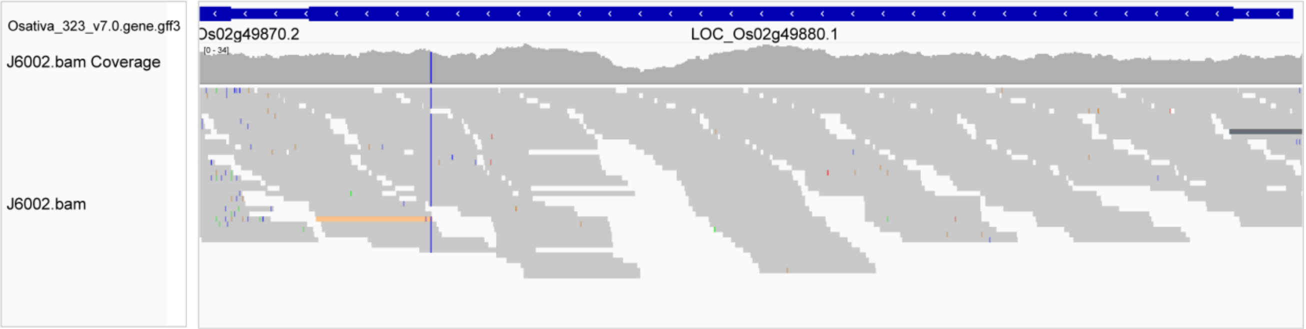

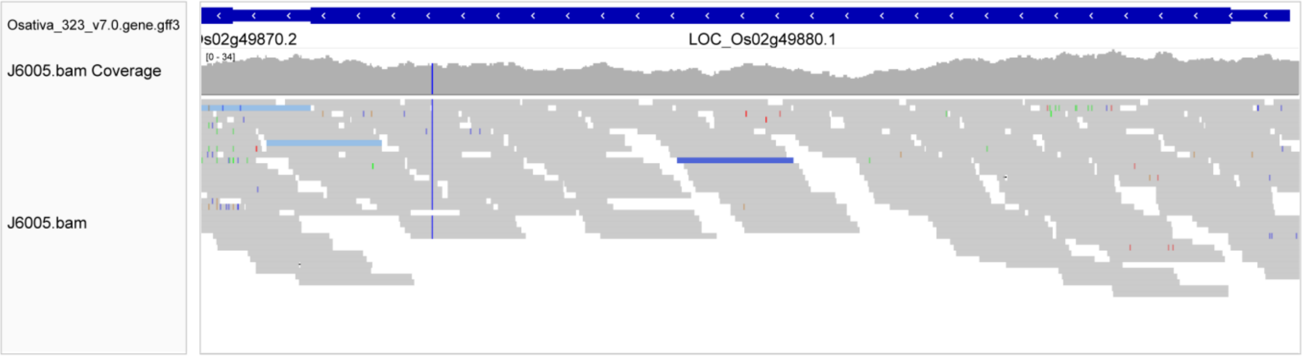

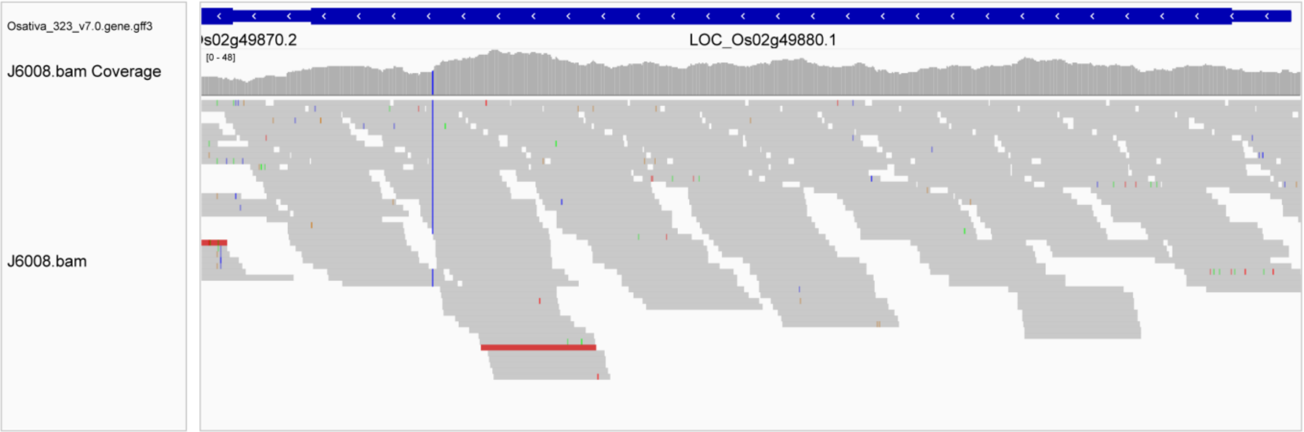

LOC_Os03g41330

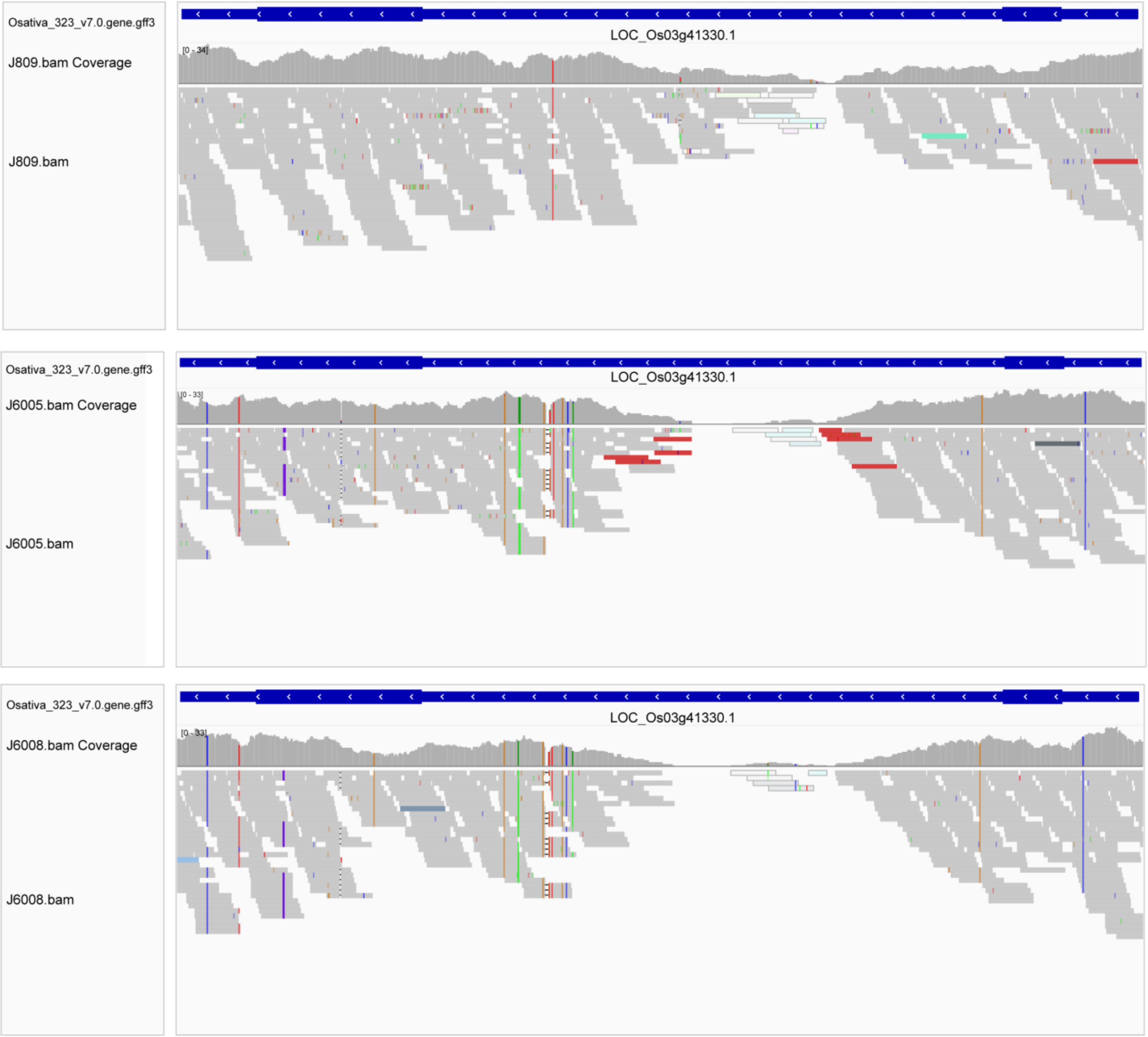

LOC_Os05g09520

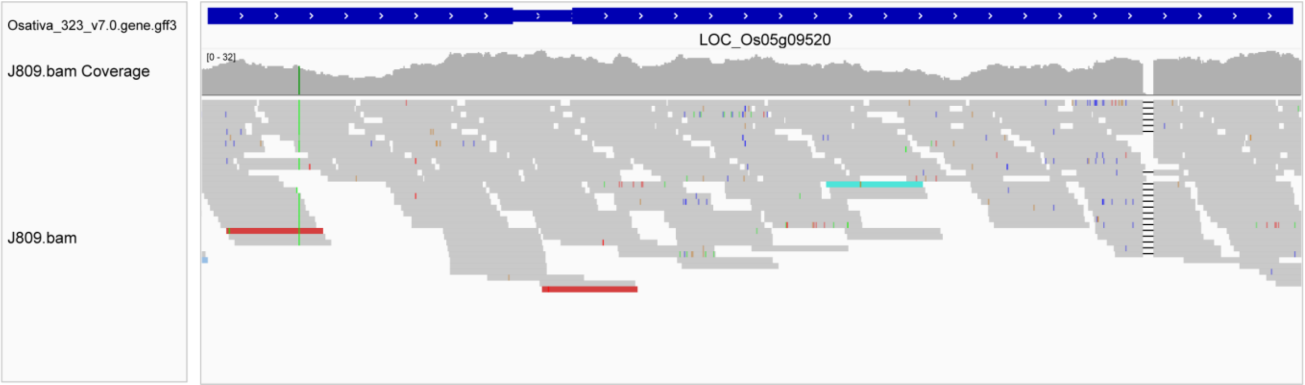
Osativa_323_v7.0.gene.gff3
J809.bam Coverage
J809.bam
LOC_Os05g09520
[0 - 32]

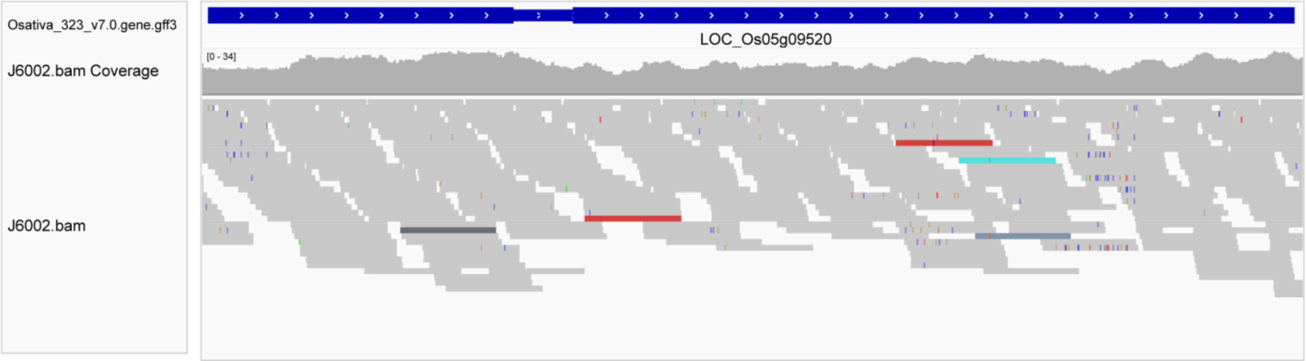
Osativa_323_v7.0.gene.gff3
J6002.bam Coverage
J6002.bam
LOC_Os05g09520
[0 - 34]

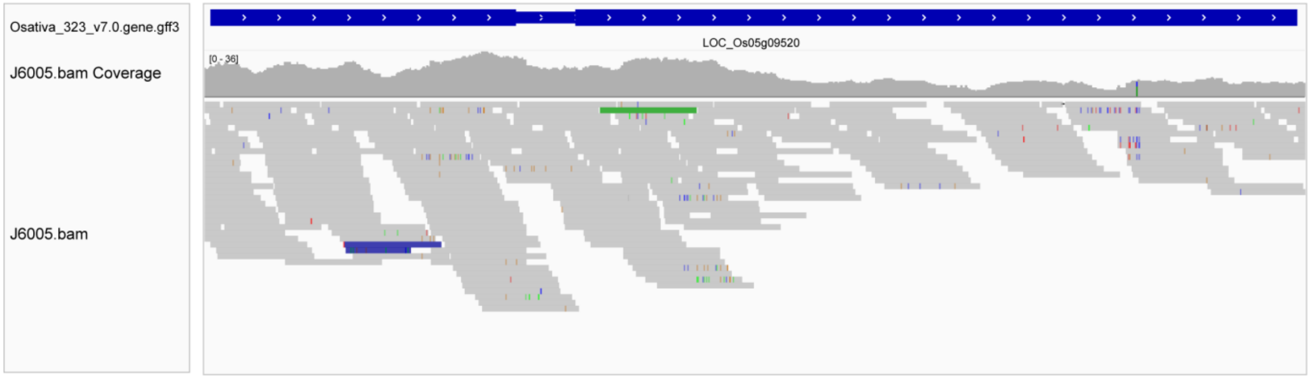
Osativa_323_v7.0.gene.gff3
J6005.bam Coverage
J6005.bam
LOC_Os05g09520
[0 - 36]

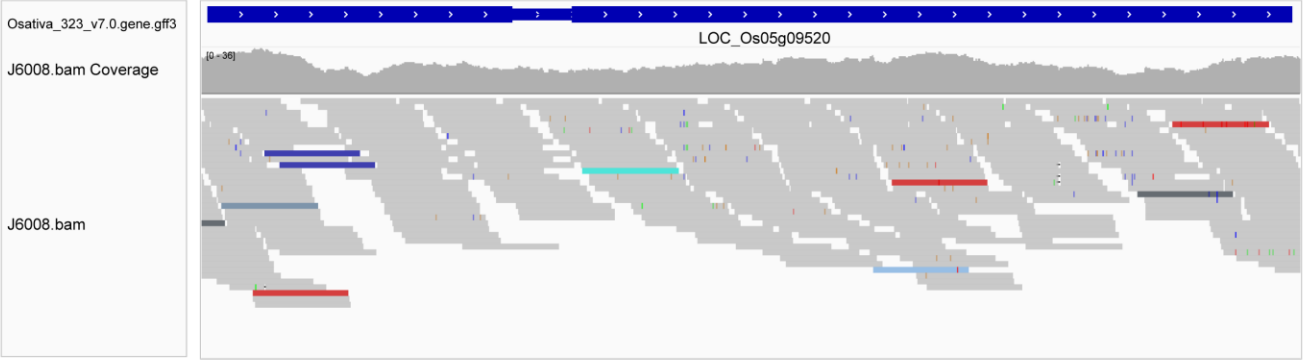
Osativa_323_v7.0.gene.gff3
J6008.bam Coverage
J6008.bam
LOC_Os05g09520
[0 - 36]

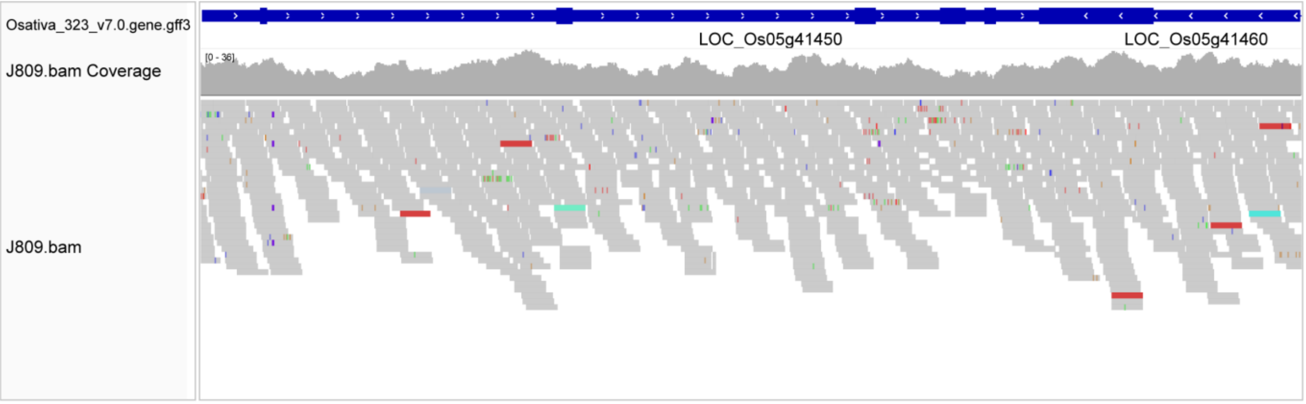

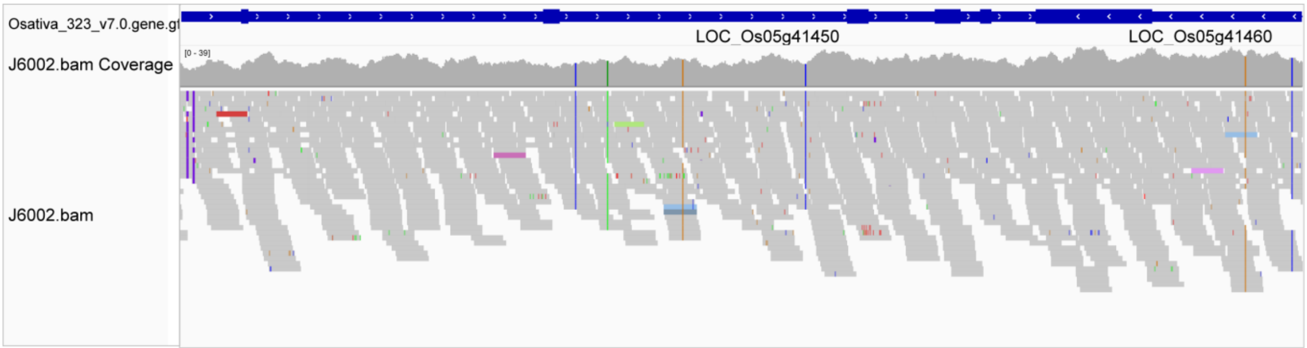

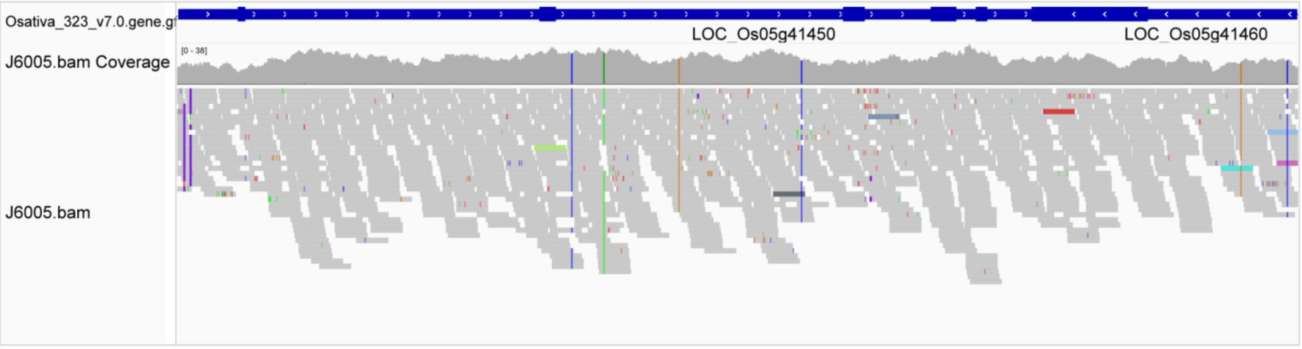

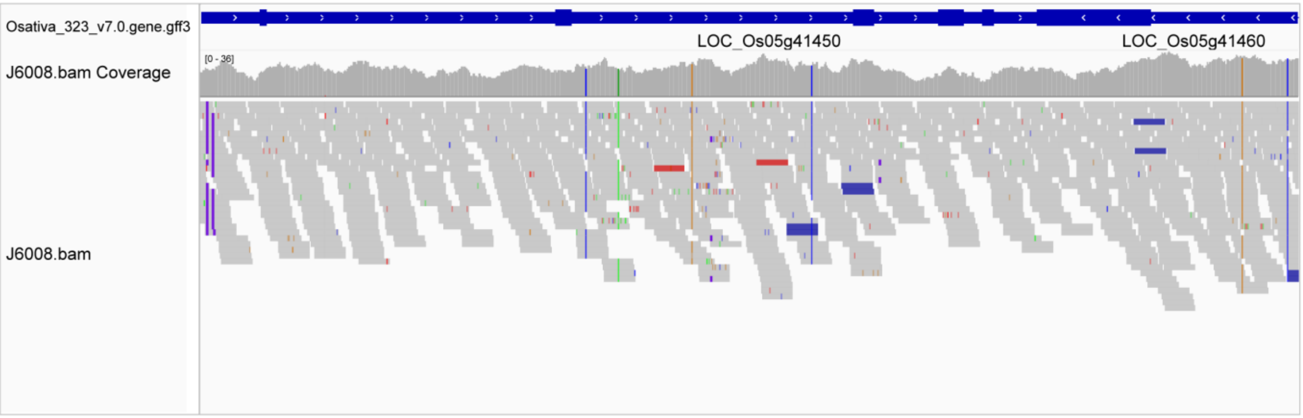

LOC_Os05g37470

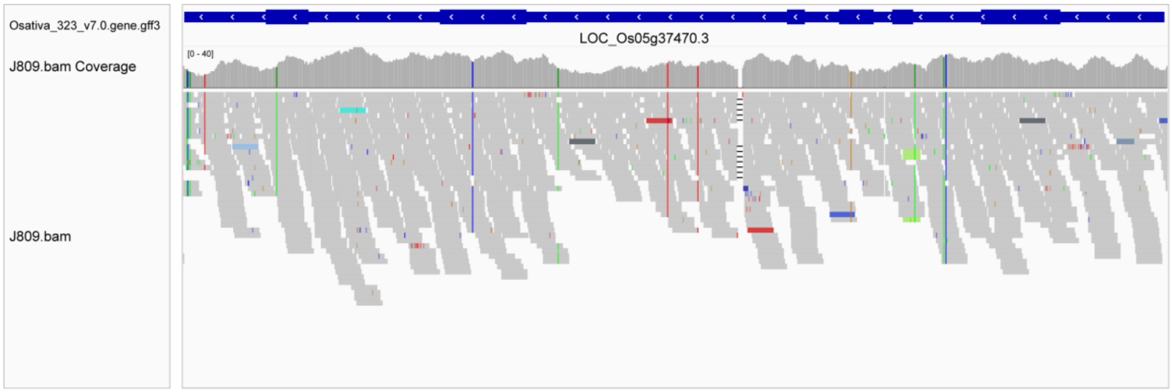

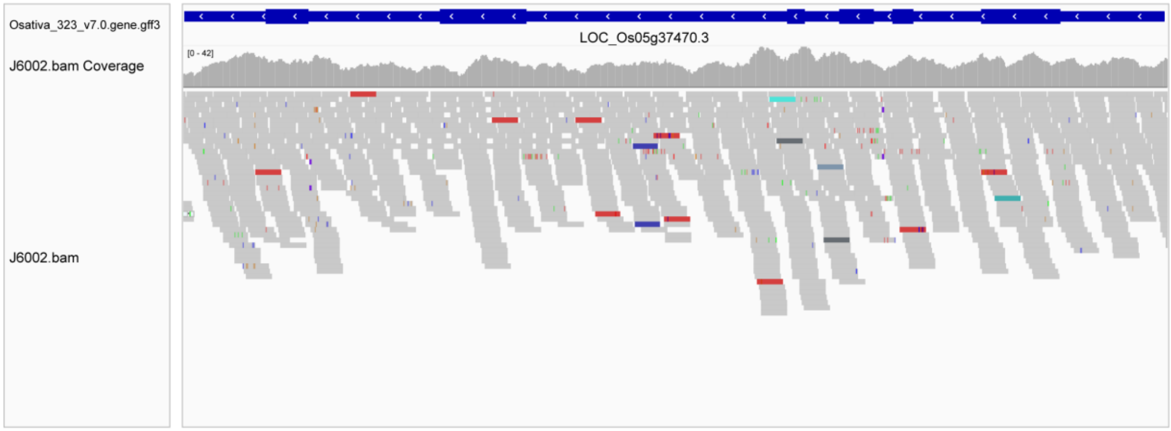

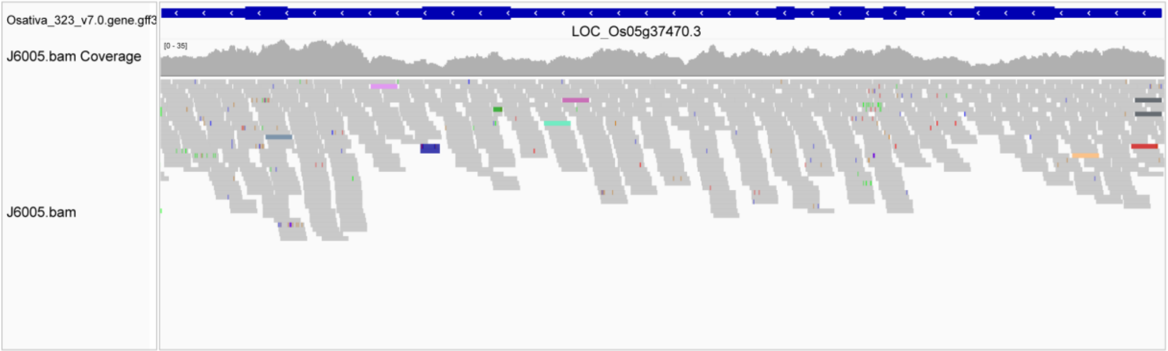

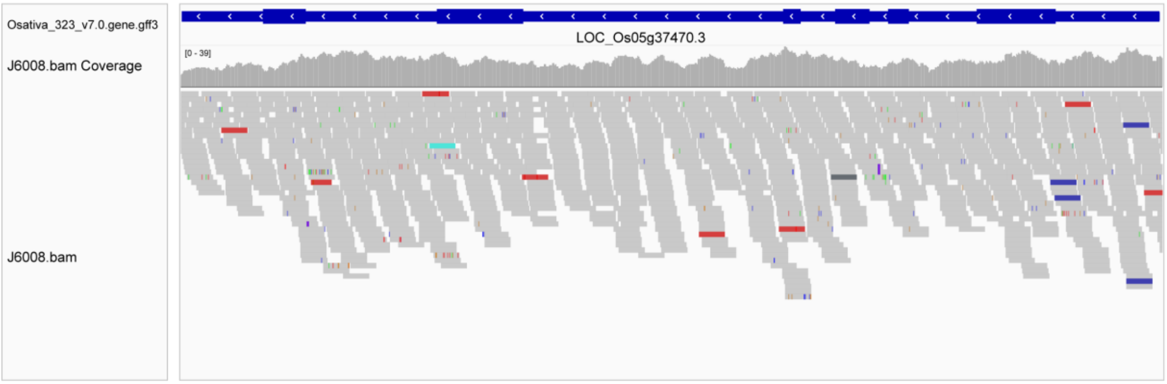

LOC_Os05g41450

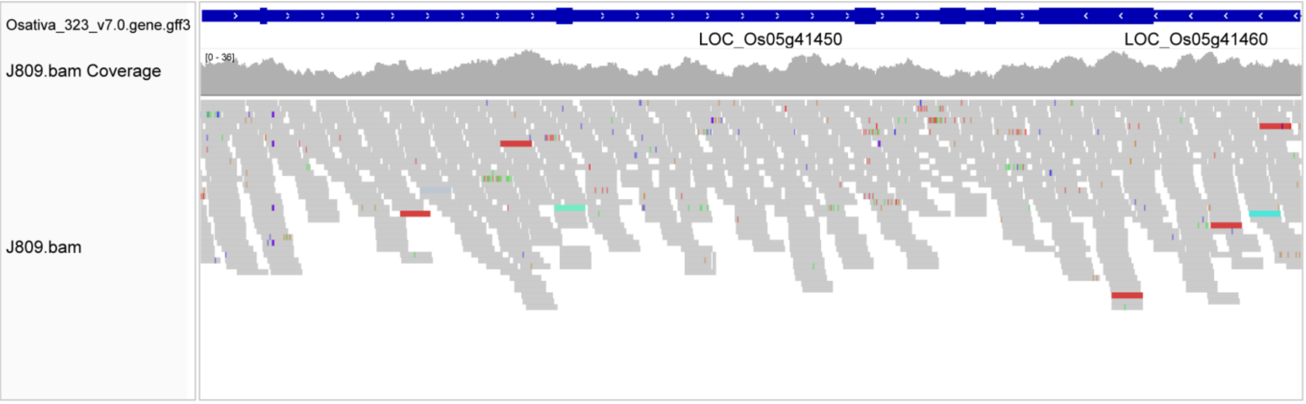

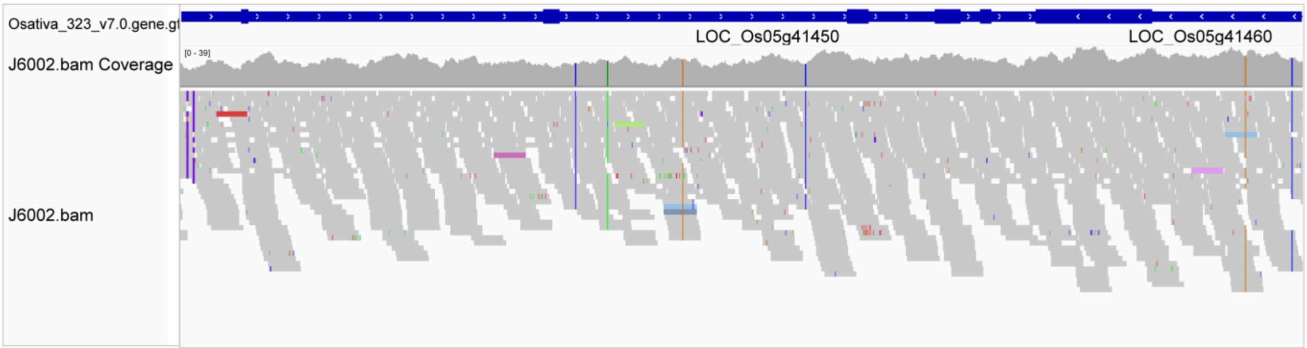

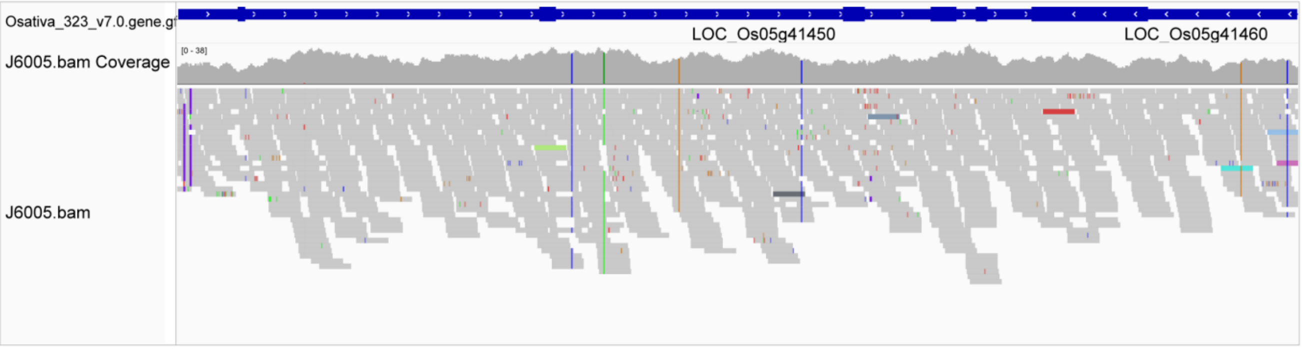

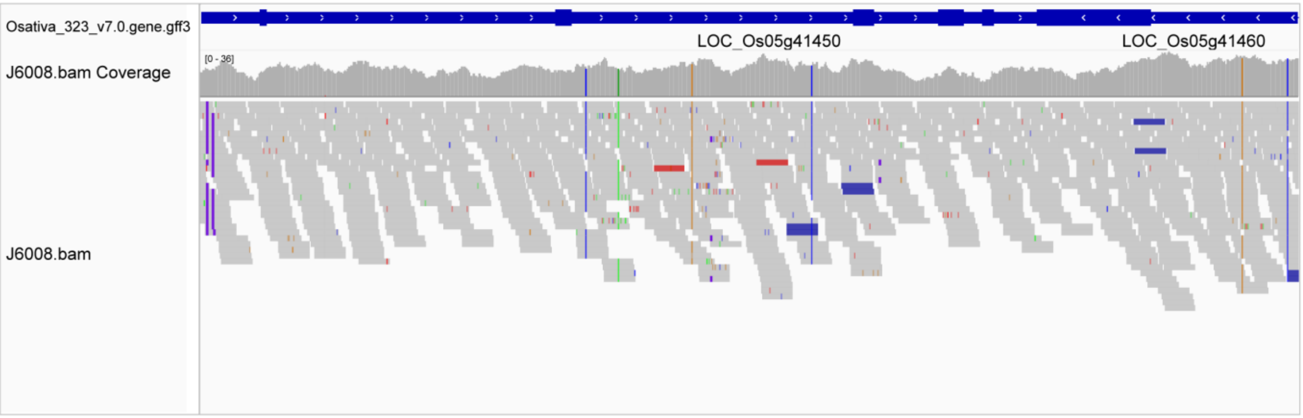

LOC_Os06g06320

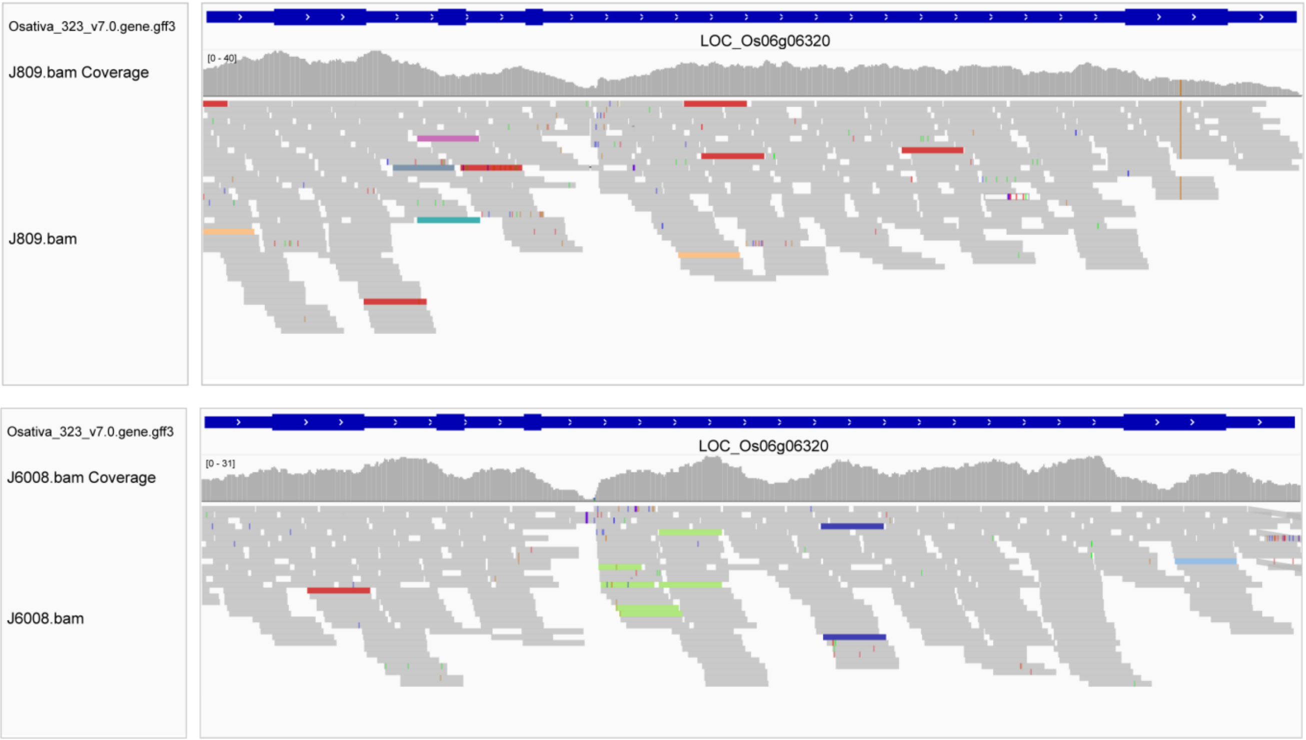
Osativa_323_v7.0.gene.gff3
J809.bam Coverage
J809.bam
LOC_Os06g06320
[0 - 40]
Osativa_323_v7.0.gene.gff3
J6008.bam Coverage
J6008.bam
LOC_Os06g06320
[0 - 31]

LOC_Os06g15620

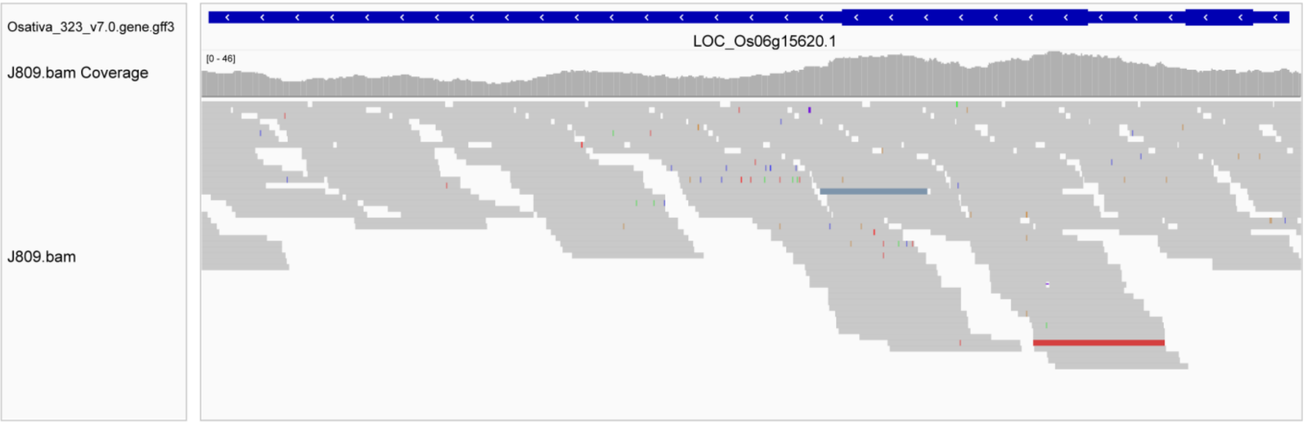

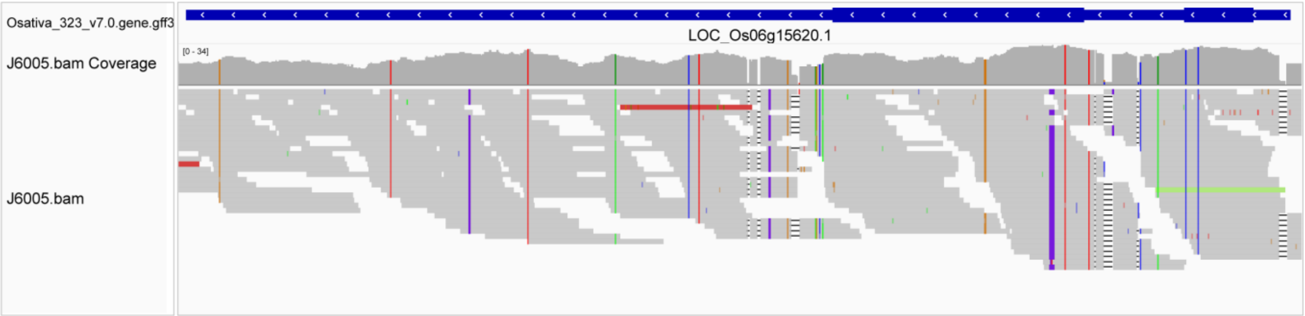

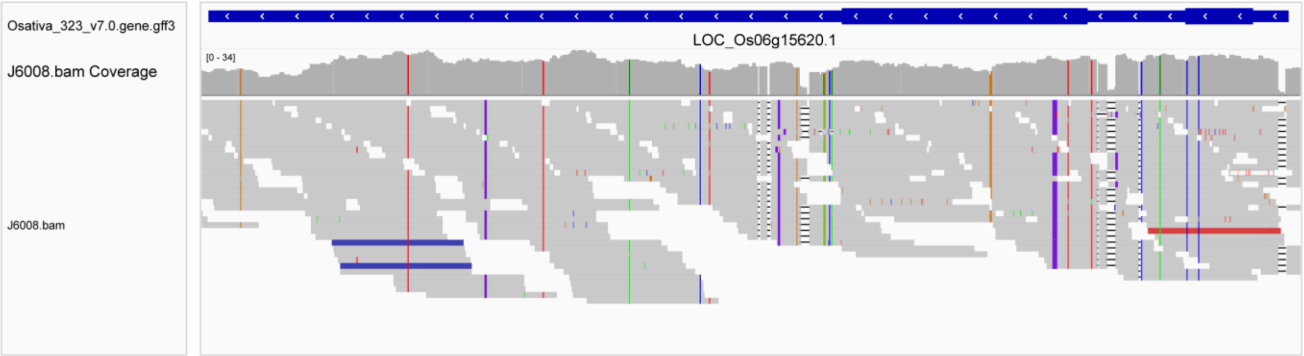

LOC_Os06g16390

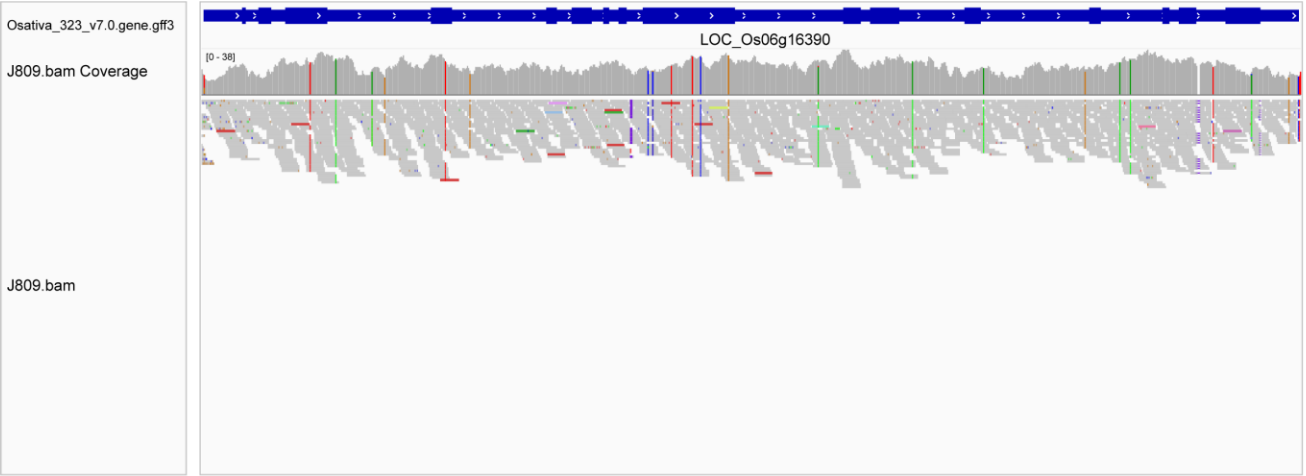

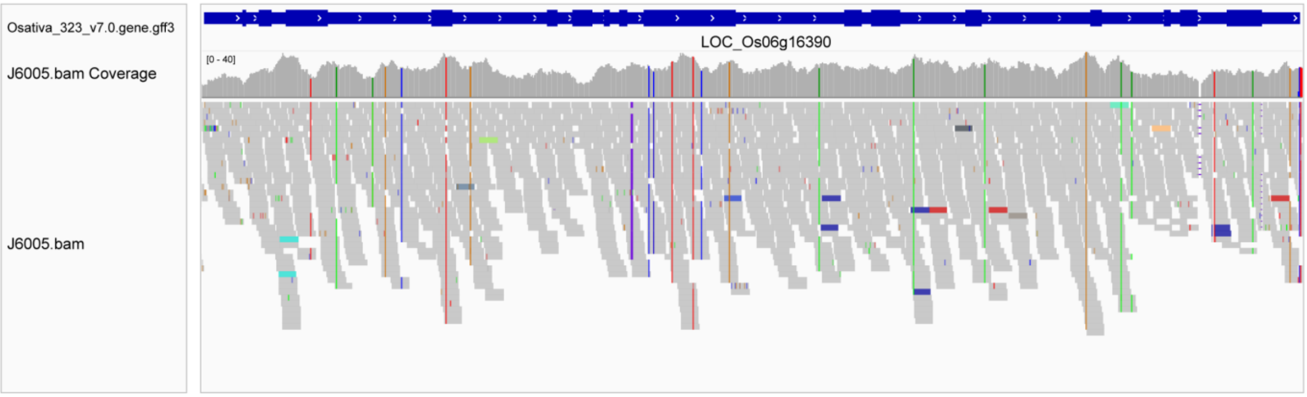

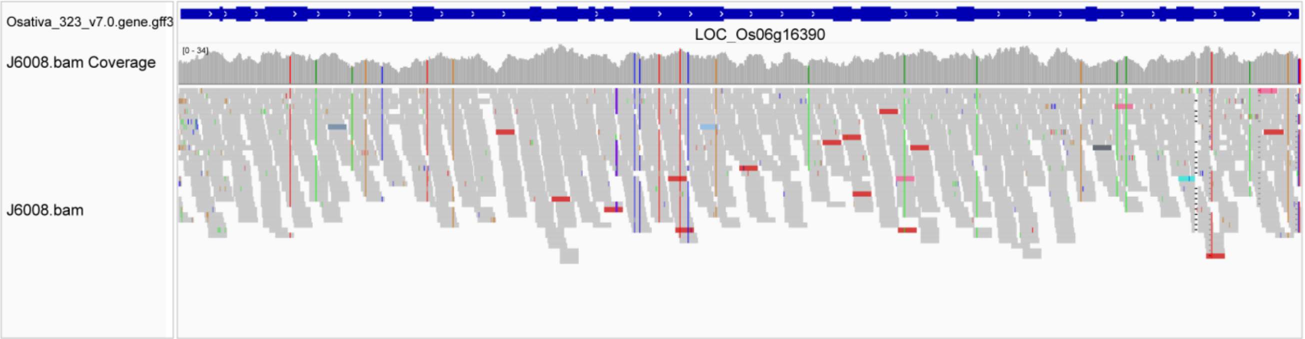

LOC_Os07g32170

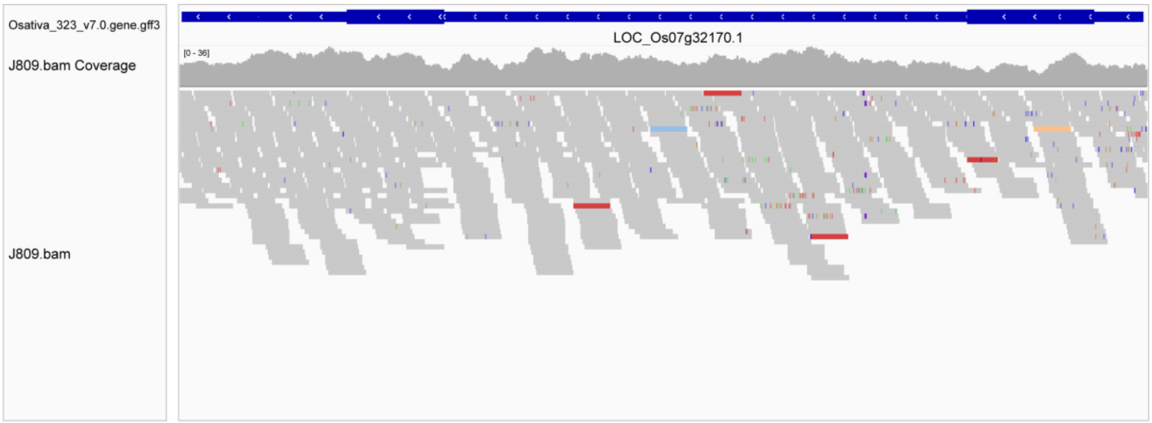

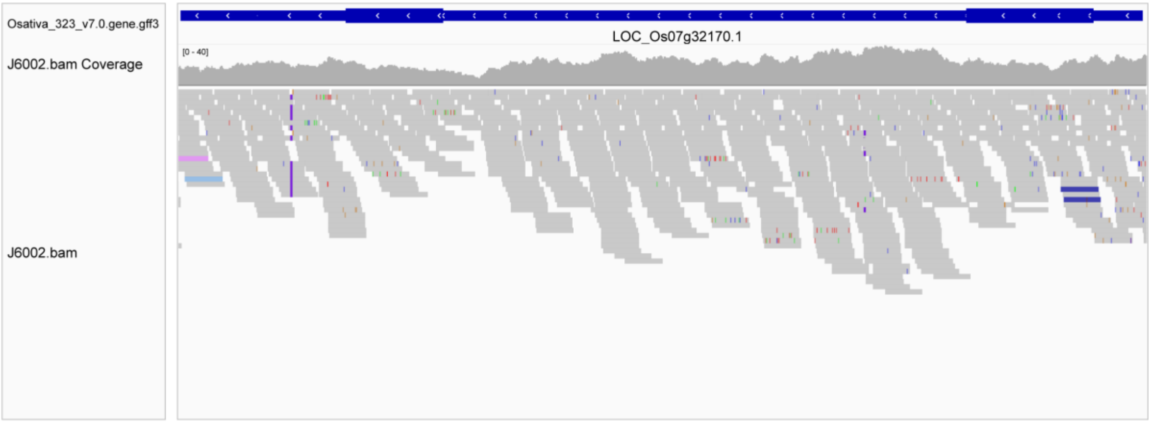

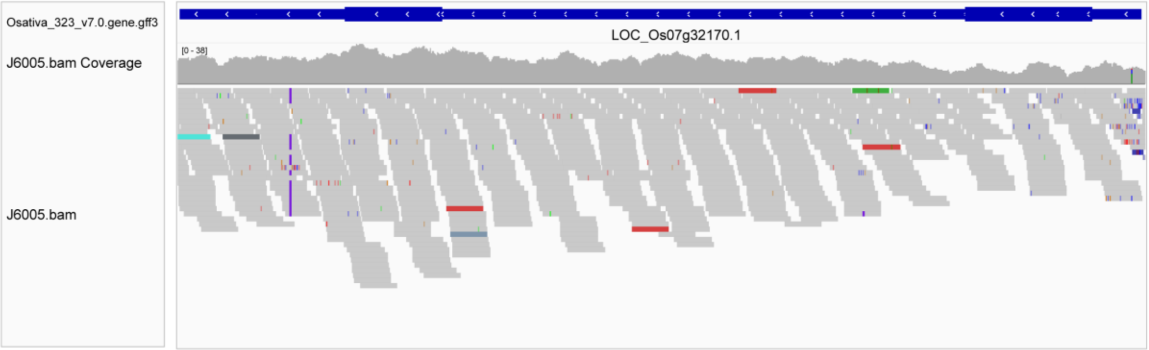

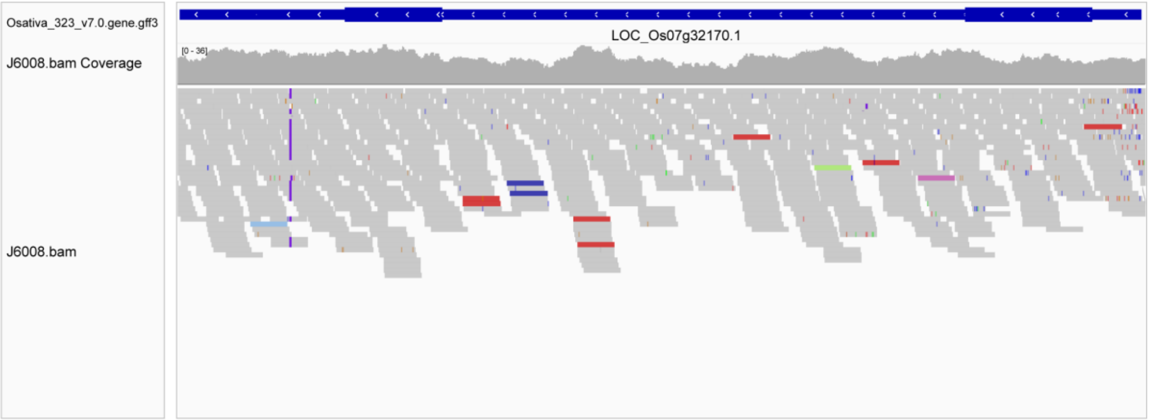

LOC_Os08g41940

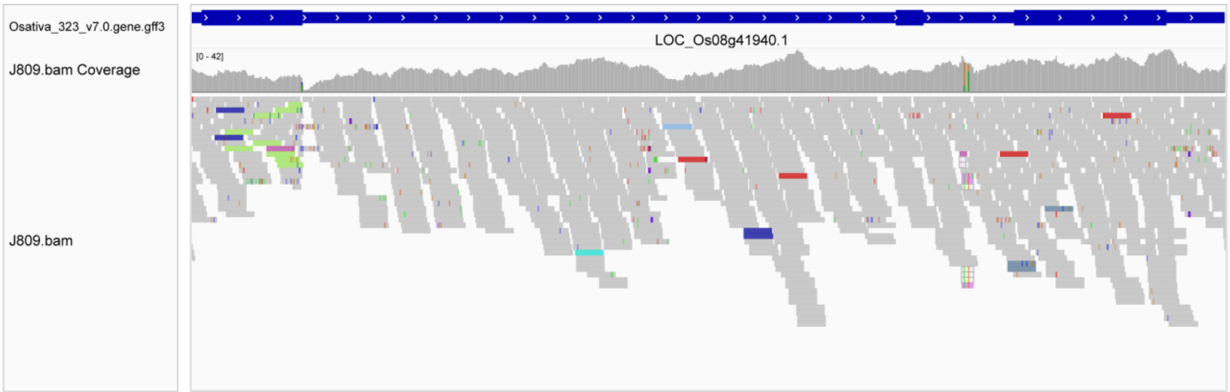
Osativa_323_v7.0.gene.gff3
J809.bam Coverage
J809.bam
LOC_Os08g41940.1
[0 - 42]

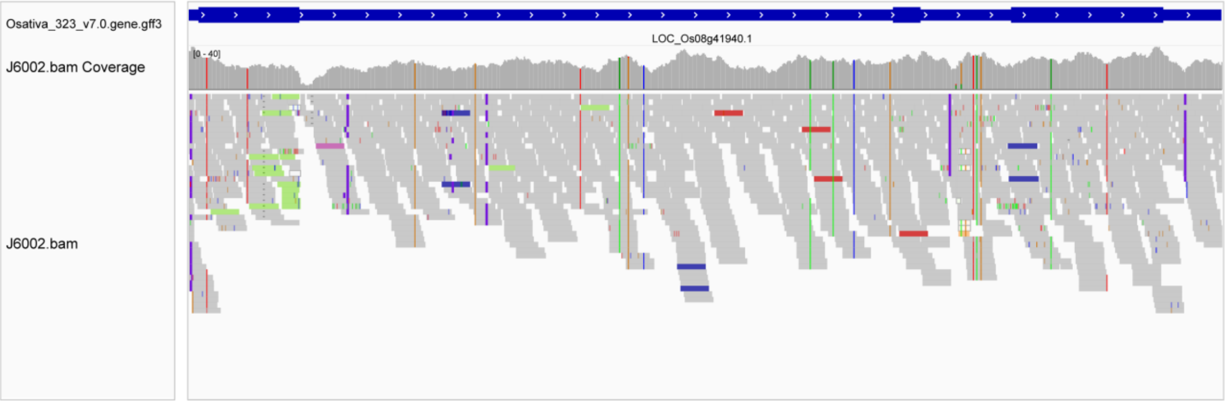
Osativa_323_v7.0.gene.gff3
J6002.bam Coverage
J6002.bam
LOC_Os08g41940.1
[0 - 40]

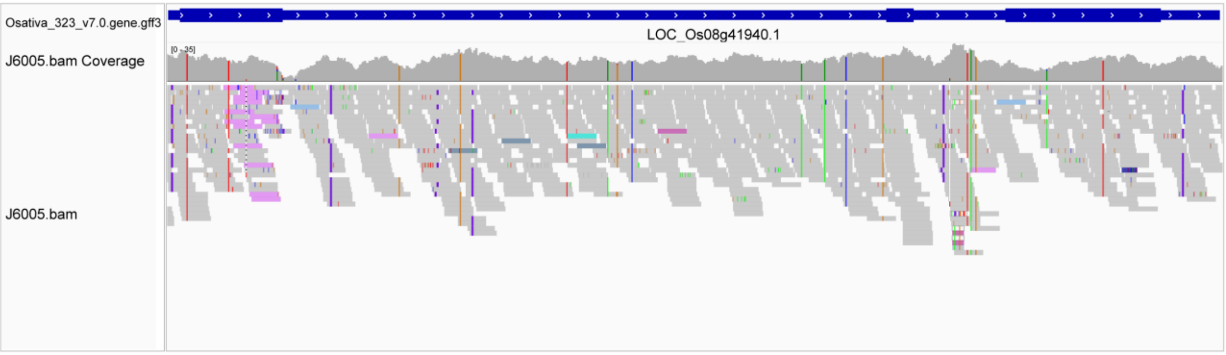
Osativa_323_v7.0.gene.gff3
J6005.bam Coverage
J6005.bam
LOC_Os08g41940.1

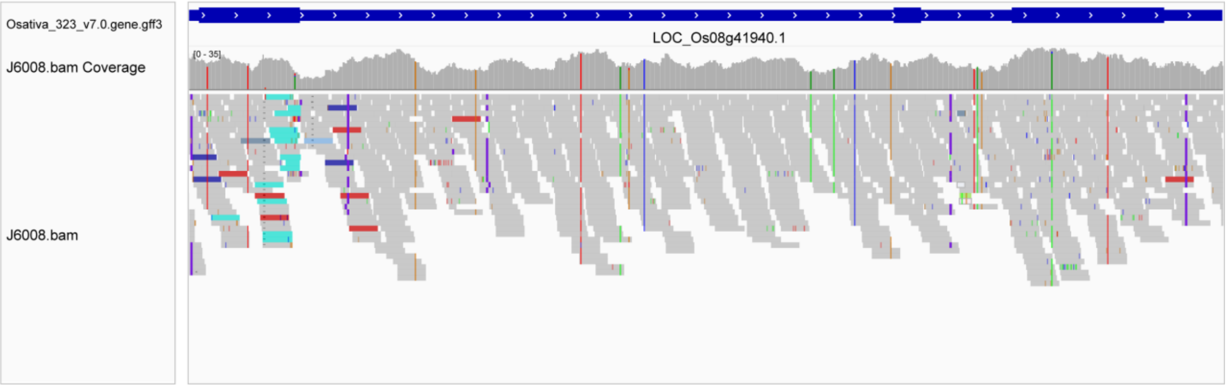
Osativa_323_v7.0.gene.gff3
J6008.bam Coverage
J6008.bam
LOC_Os08g41940.1
[0 - 35]

LOC_Os08g42440

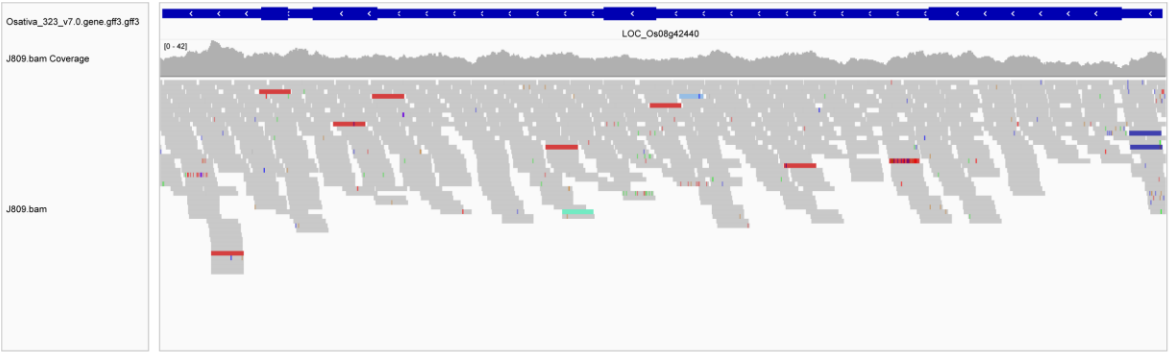

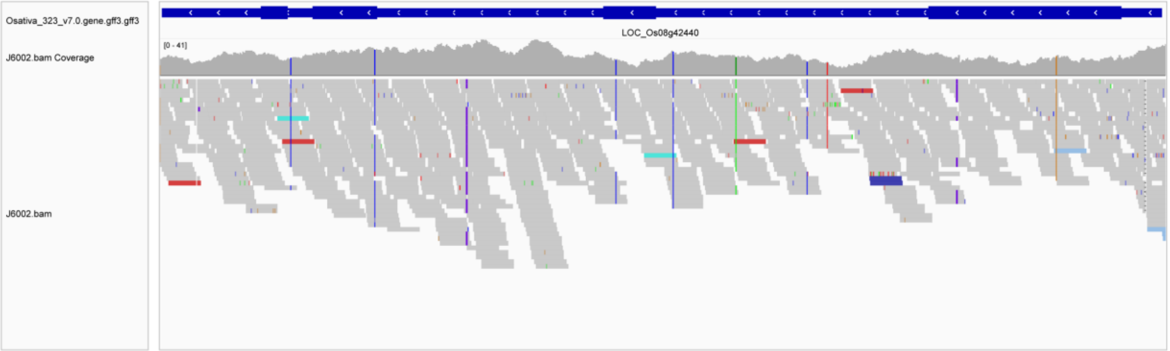

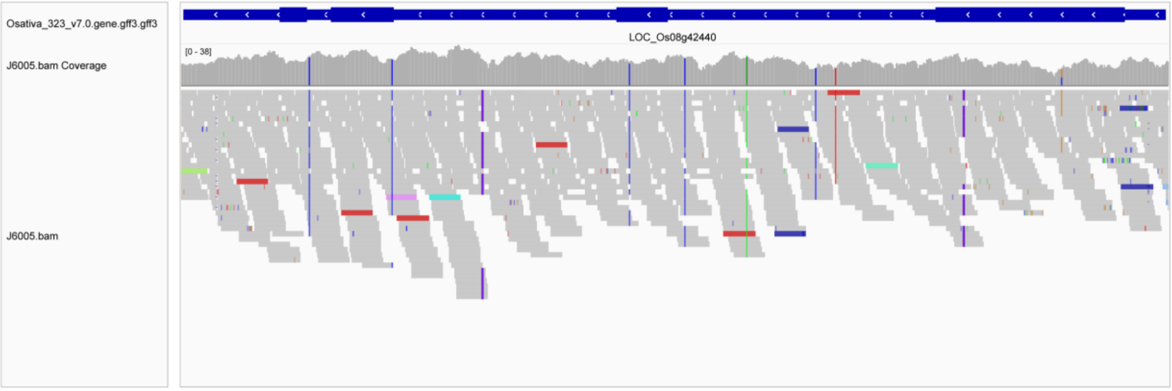

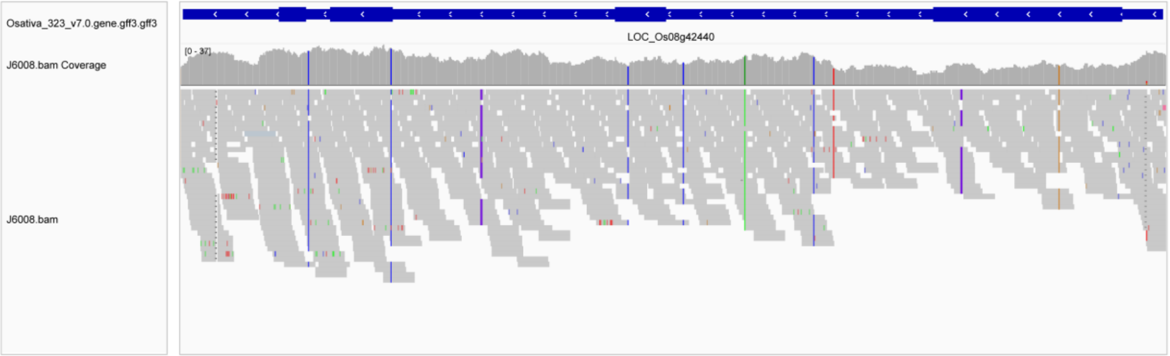

LOC_Os08g42540

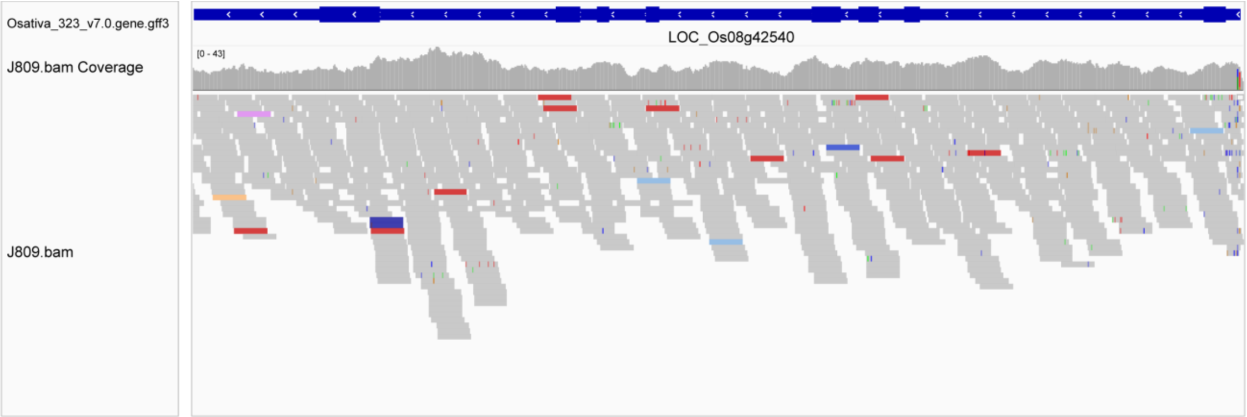

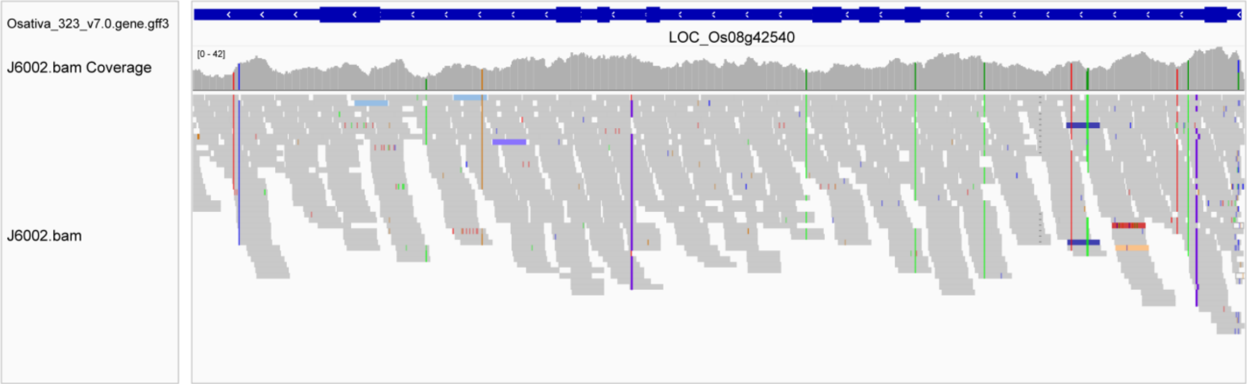

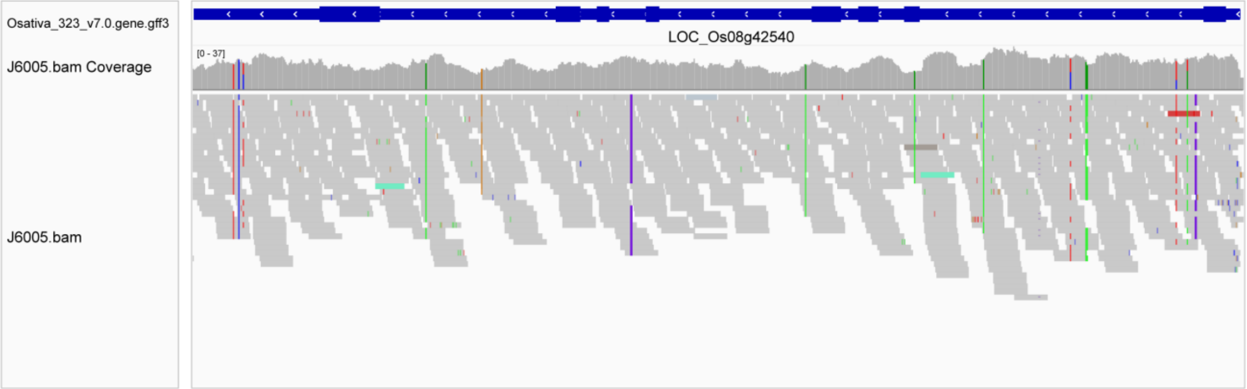

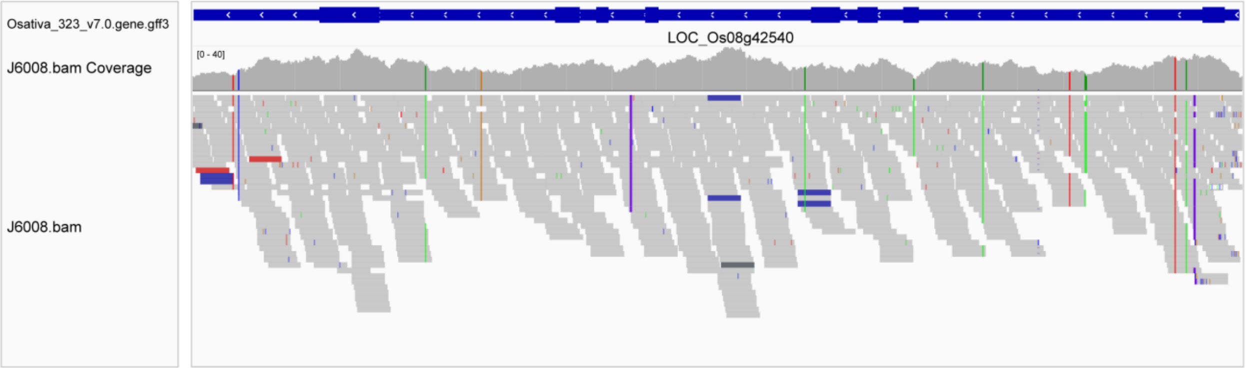

LOC_Os11g08410

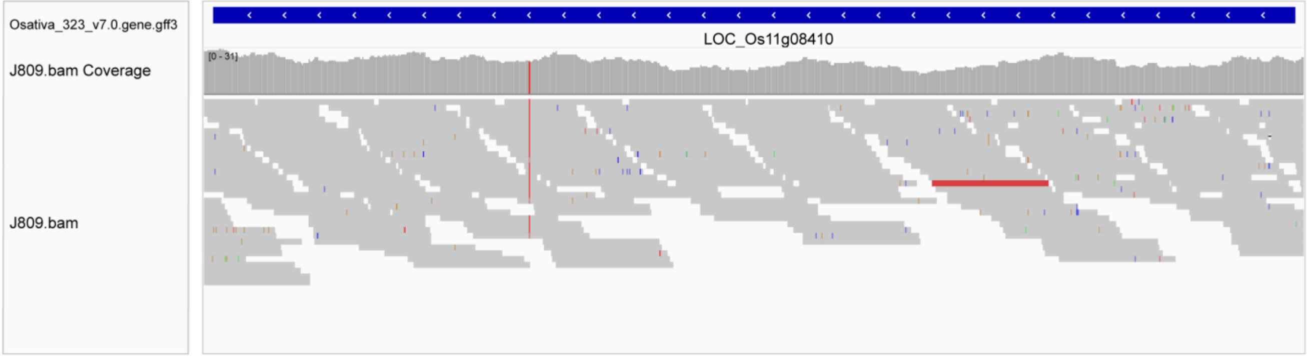

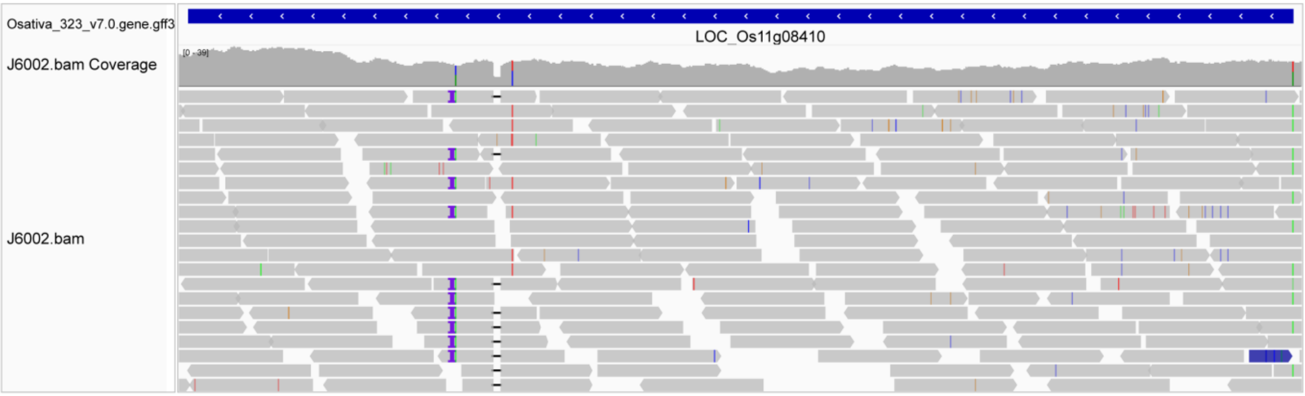

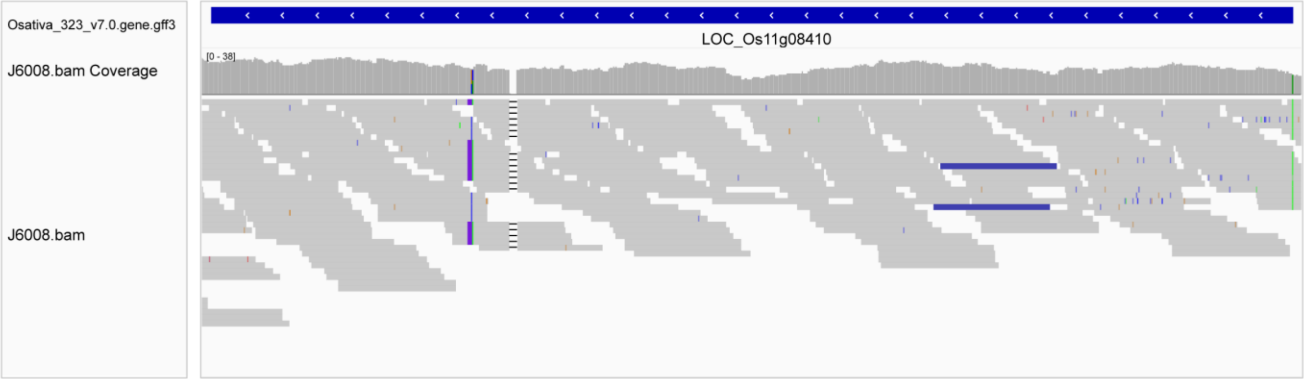

Supplement: Supplementary file 1 [file genes-14-02178-s001.zip › Supplementary_Material 3.pdf]
